# NDR2 is critical for osteoclastogenesis by regulating ULK1-mediated mitophagy

**DOI:** 10.1172/jci.insight.180409

**Published:** 2024-11-19

**Authors:** Xiangxi Kong, Zhi Shan, Yihao Zhao, Siyue Tao, Jingyun Chen, Zhongyin Ji, Jiayan Jin, Junhui Liu, Wenlong Lin, Xiao-jian Wang, Jian Wang, Fengdong Zhao, Bao Huang, Jian Chen

**Affiliations:** 1 Department of Orthopaedic Surgery, Sir Run Run Shaw Hospital, Zhejiang University School of Medicine, Hangzhou, China.; 2 Key Laboratory of Musculoskeletal System Degeneration and Regeneration Translational Research of Zhejiang Province, Hangzhou, China.; 3 Department of General Surgery, Sir Run Run Shaw Hospital, Zhejiang University School of Medicine, Hangzhou, China.; 4 Institute of Immunology and Department of Orthopedic Surgery, The Second Affiliated Hospital, Zhejiang University School of Medicine, Hangzhou, China.; 5 Department of Wound Healing, The First Affiliated Hospital of Wenzhou Medical University, Wenzhou, China.

**Keywords:** Development, Metabolism, Autophagy, Bone marrow differentiation, Osteoporosis

## Abstract

Bone homeostasis primarily stems from the balance between osteoblasts and osteoclasts, wherein an augmented number or heightened activity of osteoclasts is a prevalent etiological factor in the development of bone loss. Nuclear Dbf2-related kinase (NDR2), also known as STK38L, is a member of the Hippo family with serine/threonine kinase activity. We unveiled an upregulation of NDR2 expression during osteoclast differentiation. Manipulation of NDR2 levels through knockdown or overexpression facilitated or hindered osteoclast differentiation, respectively, indicating a negative feedback role for NDR2 in the osteoclastogenesis. Myeloid NDR2-dificient mice (*Lysm^+^NDR2^fl/fl^*) showed lower bone mass and further exacerbated ovariectomy-induced or aging-related bone loss. Mechanically, NDR2 enhanced autophagy and mitophagy through mediating ULK1 instability. In addition, ULK1 inhibitor (ULK1-IN2) ameliorated *NDR2* conditional KO–induced bone loss. Finally, we clarified a significant inverse association between NDR2 expression and the occurrence of osteoporosis in patients. The NDR2/ULK1/mitophagy axis is a potential innovative therapeutic target for the prevention and management of bone loss.

## Abstract 2

Our study has unveiled the mechanism of osteoclast differentiation mediated by the NDR2-ULK1-mitophagy axis, which has been validated in mice pathological models and osteoporosis patients.

## Introduction

The maintenance of bone mass and physiological function relies on a dynamic equilibrium between osteoclas-mediated (OC-mediated) bone resorption and osteoblast-mediated (OB-mediated) bone formation (1). OCs originate from hematopoietic stem cells within the bone marrow mononuclear cells (BMMs), with their formation and function being regulated by macrophage–colony stimulating factor (M-CSF) and receptor activator of nuclear factor-κB ligand (RANKL) (2). Notably, excessive activation of OCs plays a crucial role in the pathogenesis of diseases such as osteoporosis, rheumatoid arthritis–induced bone destruction, and cancer-related bone metastasis (3). Therefore, it is imperative to investigate the regulatory mechanisms underlying OC formation and to identify novel targets for preventing and treating disorders related to bone metabolism.

NDR2 (nuclear Dbf2-related kinase) serine/threonine kinase, also referred to as STK38L, has been identified as a novel member of the Hippo family (4). In addition to a central kinase catalytic domain, NDR2 also features a conserved N-terminal regulatory (NTR) domain and a hydrophobic motif at the C-terminus (5). NDR2 has been recognized as protein kinase that involves in a variety of biological processes, encompassing morphological alterations, centrosome duplication, cell cycle regulation, apoptosis induction, embryonic development, neurodevelopmental processes, and cancer biology (6–13). While several components of the Hippo signaling pathway, such as YAP/TAZ/TEAD complex, RASSF2, MST2, and Ajuba, are known to play crucial roles in OC differentiation (14), the specific involvement of NDR1/2 in this process remains elusive.

Autophagy is a self-clearing process and the degradation of the packaged contents within these autophagosomes facilitates recycling and renewal of cellular components necessary for metabolic updates and turnover of certain organelles (15, 16). OCs secrete β3 integrin and generate actin rings that attach to the bone surface, resulting in the formation of ruffled borders within resorption lacunae. These cells also release autophagy-associated proteins such as tartrate-resistant acid phosphatase (TRAP) and cathepsin K (CTSK), which play crucial roles in bone resorption (17). Key autophagy-related proteins including ATG5, ATG7, ATG4B, and LC3 are essential for the formation of osteoclastic ruffled borders (18, 19). Manipulating autophagic activity effectively modulates the extent of osteoclastic differentiation. TET2 facilitates OC differentiation by downregulating BCL2 expression and positively modulating BECN1-dependent autophagy (20).

The ULK1 complex, consisting of ULK1, ATG13, ATG101, and FIP200 and considered the initiator of autophagy, is activated by upstream kinases during energy or nutrient deprivation to regulate downstream substrates such as ATG9, P62, and PI3KC3-C1 complexes (21). However, there is limited research on the regulation of ULK1 in OC differentiation. Multiple posttranslational modifications, including phosphorylation, acetylation, and ubiquitination, have been demonstrated to modulate ULK1 activity, with particular emphasis on its phosphorylation (22). ULK1 can be phosphorylated by mTORC1, AMPK, and p38 MAPK-related kinases (23, 24). Our previous studies have demonstrated that NDR2 can also induce ubiquitination and degradation of retinoic acid inducible gene I (RIG-I), thereby exerting antiviral immunity (25). Recently, NDR2 was found to phosphorylate ULK1 at Ser495, which resulted in proteasomal degradation of ULK1 and inhibition of autophagy (26). The potential role of the kinase NDR2 in modulating autophagy level and affecting OC differentiation and function warrants further investigation.

We have discovered that the degradation of ULK1 is mediated by NDR2-induced phosphorylation, resulting in alterations in the expression of downstream autophagy-related proteins. This subsequently restricts the levels of autophagy and mitophagy within OCs, consequently affecting OC differentiation and bone resorption. Our findings hold significant implications for further investigating the role of kinase phosphorylation and ULK1-mediated autophagy in osteoporosis-related diseases, while also offering targets and a theoretical foundation for preventing and treating bone metabolic disorders.

## Results

### NDR2 inhibited osteoclastogenesis and had little effect on osteogenesis.

**Figure 1:**
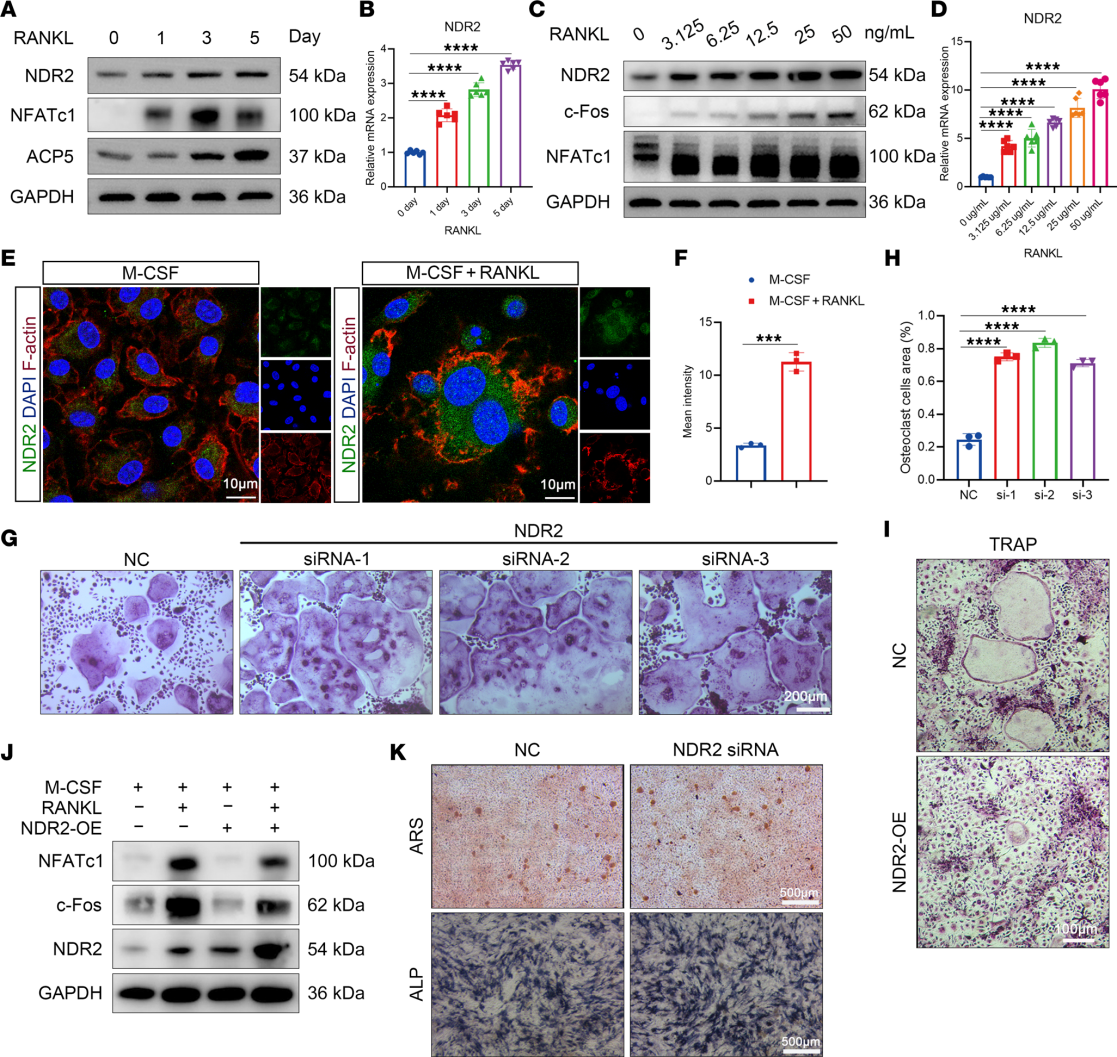
NDR2 inhibited osteoclastogenesis and had little effect on osteogenesis. (**A**) Proteins were extracted from BMMs at different time points of osteoclast induction, and Western blot was performed. The concentrations of M-CSF and RANKL used were 50 ng/mL and 25 ng/mL, respectively. (**B**) The mRNA levels of the NDR2 were assessed at various time points during osteoclast differentiation using qPCR (*n* = 6). (**C**) NDR2 protein expression was examined in BMMs treated with different concentrations of RANKL for 48 hours. (**D**) qPCR analysis was performed to assess the mRNA levels of the NDR2 after 48 hours of treatment with varying concentrations of RANKL (*n* = 6). (**E**) Representative images of NDR2 fluorescence staining were obtained, with NDR2 shown in green, F-actin in red, and DAPI in blue. (**F**) Statistical analysis was conducted to evaluate the intensity of NDR2 fluorescence (*n* = 3). (**G**) BMMs were transfected with NDR2 siRNA for 12 hours, followed by induction treatment with RANKL (25 ng/mL) for 5 days and subsequent TRAP staining. (**H**) Statistical analysis was performed on the area of TRAP^+^ osteoclasts (*n* = 3). (**I**) BMMs were subjected to TRAP staining 5 days after RANKL stimulation. (**J**) Western blotting was conducted on MC3T3-E1 cells that were subjected to a 7-day period of osteogenic induction in order to evaluate the protein levels associated with bone formation. (**K**) Alizarin red staining was used to visualize mineralized nodules after 21 days of osteogenic induction, while alkaline phosphatase staining was utilized to detect early-stage osteoblastic differentiation after 14 days. Statistical analyses were determined by 2-tailed Student’s *t* test (**F**) or 1-way ANOVA (**B**, **D**, and **H**). ***P < 0.001, ****P < 0.0001. Data were presented as mean ± SD.

To elucidate the role of NDR2 in osteoclastogenesis, we initially assessed the expression level of NDR2 during OC differentiation. Both protein and gene expression levels of NDR2 were found to be upregulated in response to OC differentiation (Figure 1, A and B, and Supplemental Figure 1A; supplemental material available online with this article; https://doi.org/10.1172/jci.insight.180409DS1) in a concentration-dependent manner (Figure 1, C and D, and Supplemental Figure 1B). Cellular immunofluorescence staining revealed an increase in fluorescence intensity of NDR2 following RANKL stimulation (Figure 1, E and F), suggesting that elevated levels of NDR2 may play a crucial role in osteoclastogenesis. In order to further ascertain whether NDR2 acts as a driving force or inhibitory factor for OC differentiation, BMMs were treated with NDR2 siRNA (Supplemental Figure 1, C and D). The group treated with NDR2 siRNA exhibited a higher number and larger area occupied by OCs compared with the control group (Figure 1, G and H). Considering the superior knockdown efficiency achieved by using NDR2 siRNA-1, subsequent experiments were conducted employing this siRNA. Consistent with TRAP staining results, Western blot demonstrated significant upregulation of OC-related markers (*Nfatc1*, *c-Fos*) upon knockdown of NDR2 (Supplemental Figure 1E). Furthermore, BMMs transfected with lentivirus overexpressing NDR2 displayed notable capacity for inhibiting osteoclastogenesis (Figure 1, I and J). These findings indicate that, while there is an initial increase in expression levels of NDR2 during OC differentiation, subsequent negative feedback mechanisms inhibit further progression toward mature osteoclastic phenotypes.

To eliminate the influence of NDR2 on OBs, we employed the MC3T3-E1 cell line for inducing osteogenic differentiation. Knockdown of NDR2 exhibited a marginal effect on osteoblastogenesis-related genes (*Runx2*, *Alpl*, *Bglap*, *Spp1*) and proteins (RUNX2) (Supplemental Figure 1, F and G), while alkaline phosphatase (ALP) and alizarin red S (ARS) staining revealed no substantial differences between the experimental groups (Figure 1K). In conclusion, NDR2 exerts negative feedback inhibition on osteoclastogenesis without affecting OBs.

### Lysm^+^NDR2^fl/fl^ mice exhibited decreased bone mass and enhanced osteoclastogenesis.

**Figure 2:**
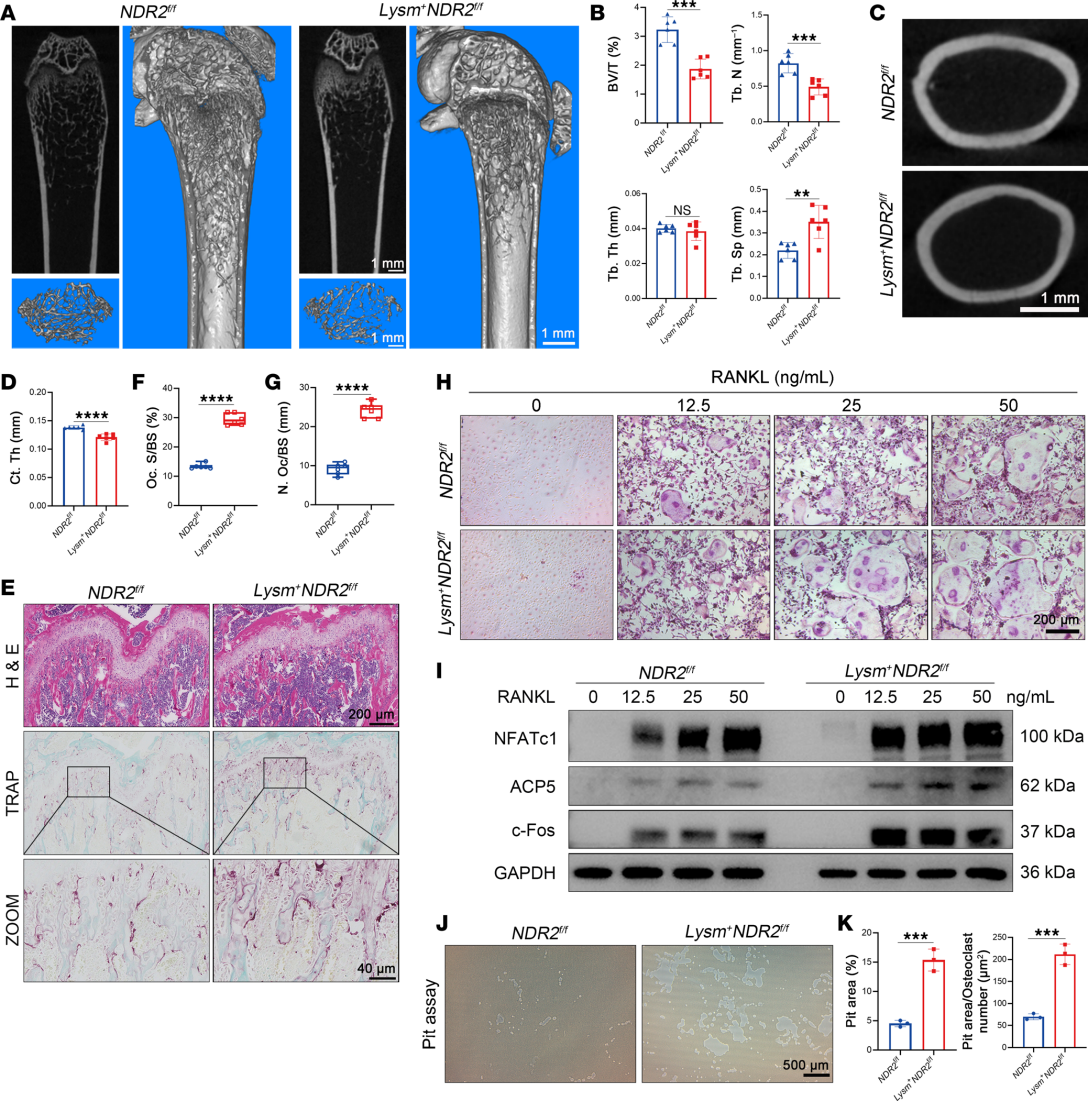
*Lysm^+^NDR2^fl/fl^* mice exhibited decreased bone mass and enhanced osteoclastogenesis. (**A**) Femurs from 2-month-old *Lysm^–^NDR2^fl/fl^* and *Lysm^+^NDR2^fl/fl^* littermates were imaged using μCT techniques. (**B**) Parameters related to trabecular bone in the proximal femur (*n* = 6) were analyzed, including bone volume to tissue volume (BV/TV), trabecular thickness (Tb.Th), trabecular number (Tb.N), and trabecular separation (Tb.Sp). (**C**) Representative 2D images of cortical bone were captured. (**D**) Statistical analysis was performed on cortical bone thickness (Ct.Th). (**E**) H&E staining and TRAP staining were conducted on sections of the femur. (**F** and **G**) Histomorphological analysis was carried out on TRAP staining, specifically osteoclast number per unit of bone surface area (N.Oc/BS) and osteoclast surface area per unit of bone surface area (Oc.S/BS; *n* = 6 per group). (**H**) Bone marrow–derived macrophages (BMMs) extracted from *Lysm^–^NDR2^fl/fl^* and *Lysm^+^NDR2^fl/fl^* littermates were induced for osteoclast differentiation at different concentrations of RANKL. After 5 days, TRAP staining was performed. (**I**) Western blotting was used to detect differences in expression levels of osteoclast-related proteins between the 2 groups after RANKL stimulation. (**J**) The activity of osteoclasts was assessed using hydroxyapatite-coated plates. (**K**) Statistical analysis was conducted to assess the extent of bone erosion area (*n* = 3). Statistical analyses were determined by 2-tailed Student’s *t* test (**B**, **D**, **F**, **G**, and **K**). **P < 0.01, ***P < 0.001, and ****P < 0.0001. Data were presented as mean ± SD.

The *Lysm^+^NDR2^fl/fl^* mice were generated by crossing *NDR2^fl/fl^* mice with *Lysm-cre* mice. The μ-CT results reveal a decrease in bone mass in *Lysm^+^NDR2^fl/fl^* mice compared with *NDR2^fl/fl^* counterparts. Specifically, there was a reduction observed in the bone volume/tissue volume (BV/TV) and trabecular number (Tb.N), accompanied by an increase in trabecular separation (Tb.Sp) (Figure 2, A and B). Additionally, conditional KO of NDR2 resulted in a significant reduction in cortical bone thickness (Ct.Th) (Figure 2, C and D). Similar observations of spontaneous bone loss were observed in both trabecular and cortical bone for female mice, which mirrored the findings seen in male mice (Supplemental Figure 2, A–D). TRAP staining revealed an increased presence of OCs in distal femoral sections of *Lysm^+^NDR2^fl/fl^* mice, and quantitative histomorphological analysis demonstrated upregulation of both the ratios of OC area/bone tissue area (Oc.S/BS) and OC number/bone tissue area (Oc.N/BS) (Figure 2, E–G).

To further elucidate whether the reduced bone mass observed in *Lysm^+^NDR2^fl/fl^* mice resulted from enhanced osteoclastogenesis, bone marrow–derived macrophages (BMMs) derived from *NDR2^fl/fl^* and *Lysm^+^NDR2^fl/fl^* mice were isolated for subsequent in vitro investigations. Under different RANKL concentrations, OCs derived from NDR2-KO mice appeared earlier and fused more, especially at lower concentrations (Figure 2H). The expression levels of OC differentiation–related markers (*Nfatc1*, *Acp5*, and *c-Fos*) were significantly higher in comparison with those observed in *NDR2^fl/fl^*-derived BMMs (Figure 2I). The demineralization assay indicated enhanced OC activity and a larger area of bone plate erosion following NDR2 knockdown (Figure 2, J and K). Consistent with these findings, there was also a propensity toward proosteoclast activity at the genetic level, exhibiting significant genetic differences even without RANKL induction (Supplemental Figure 2E). Therefore, it can be inferred that NDR2 knockdown accelerates bone loss by promoting osteoclastogenesis and enhancing OC activity.

### Lysm^+^NDR2^fl/fl^ mice aggravated OVX-induced and aging-related bone loss.

**Figure 3:**
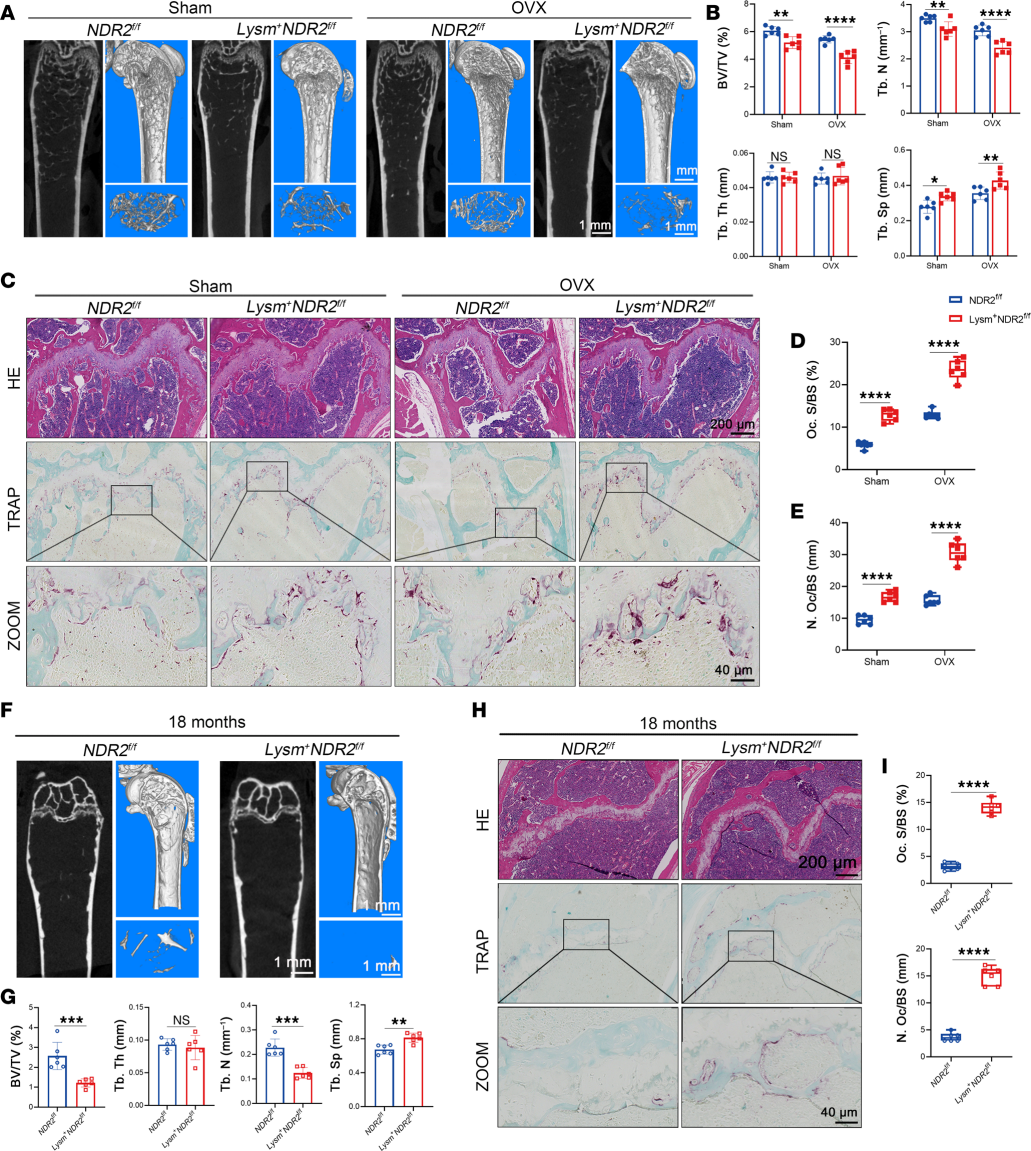
*Lysm^+^NDR2^fl/fl^* mice aggravated OVX-induced and aging-related bone loss. (**A**) OVX was performed on 2-month-old *Lysm^–^NDR2^fl/fl^* and *Lysm^+^NDR2^fl/fl^* mice, and the mice were sacrificed after 8 weeks for femur sample collection. μCT was utilized to illustrate the alterations in femoral trabeculae. (**B**) Analysis of parameters associated with trabecular bone in the proximal femur was conducted (*n* = 6). (**C**) H&E staining and TRAP staining were performed on sections of the femur. (**D** and **E**) Histomorphological analysis of TRAP staining was carried out (*n* = 6). (**F**) Representative μCT images of 18-month-old *Lysm^–^NDR2^fl/fl^* and *Lysm^+^NDR2^fl/fl^* mouse femurs. (**G**) Analysis of parameters related to trabecular bone in the proximal femur (*n* = 6). (**H**) H&E staining and TRAP staining were conducted on sections of the femur. (**I**) Histomorphological analysis of TRAP staining (*n* = 6). Statistical analyses were determined by 2-tailed Student’s *t* test (**G** and **I**), and 2-way ANOVA (**B**, **D**, and **E**). ns indicated no statistical difference, *P < 0.05, **P < 0.01, ***P < 0.001, and ****P < 0.0001. Data were presented as mean ± SD.

Osteoporosis is a prevalent complication in postmenopausal women, with estrogen withdrawal being the primary factor contributing to OC hyperactivity (27). To investigate the potential involvement of NDR2 in estrogen deficiency–induced osteoporosis, ovariectomy was performed on 12-week-old female *Lysm^+^NDR2^fl/fl^* mice. Surprisingly, μCT 2D and 3D images reveal a significant reduction in bone trabeculae after OVX in the *Lysm^+^NDR2^fl/fl^* group compared with the *NDR2^fl/fl^* group, as evidenced by a decrease in BV/TV (%) and Tb.Th (mm^–1^) and an increase in Tb.Sp (mm) (Figure 3, A and B). The histomorphological staining results further confirm a significant increase in the number of OCs in vivo following NDR2 KO (Figure 3, C–E), indicating that NDR2 exacerbates osteoclastogenesis and promotes bone loss after OVX modeling. Osteocalcin (OCN), a noncollagenous protein expressed and secreted by OBs, serves as an indicator of bone formation in vivo. OCN fluorescence staining revealed no significant difference in OB numbers between groups, indicating that there was no effect on bone formation (Supplemental Figure 3, A and B).

Aging is also a significant contributing factor to the development of osteoporosis (28, 29); however, its underlying mechanism remains intricate and not yet fully elucidated. In the 18-month *Lysm^+^NDR2^fl/fl^* group compared with *NDR2^fl/fl^* group, there was observed a decrease in bone mass (Figure 3, F and G, and Supplemental Figure 3, E and F), accompanied by an increased number of OCs (Figure 3, H and I). These findings suggest that NDR2 KO further exacerbates age-related bone loss. Furthermore, we discovered a significant downregulation of NDR2 expression levels in both the OVX model and aging groups (Supplemental Figure 3, C, D, G, and H), indicating that the diminished inhibitory effect of NDR2 on osteoclastogenesis may serve as an important factor in the pathogenesis of osteoporosis.

### NDR2 regulated osteoclastogenesis via ULK1.

To further elucidate the underlying mechanisms of NDR2 in regulating OC differentiation, we initially investigated the classical pathway of osteoclastogenesis. Following RANKL induction, there was a marginal alteration in MAPK/NF-κB levels upon NDR2 knockdown, with even slight downregulation observed in Erk and p38 phosphorylation levels. These findings suggest that the augmented osteoclastogenesis resulting from NDR2 knockdown is not mediated through these canonical pathways (Supplemental Figure 4A).

**Figure 4:**
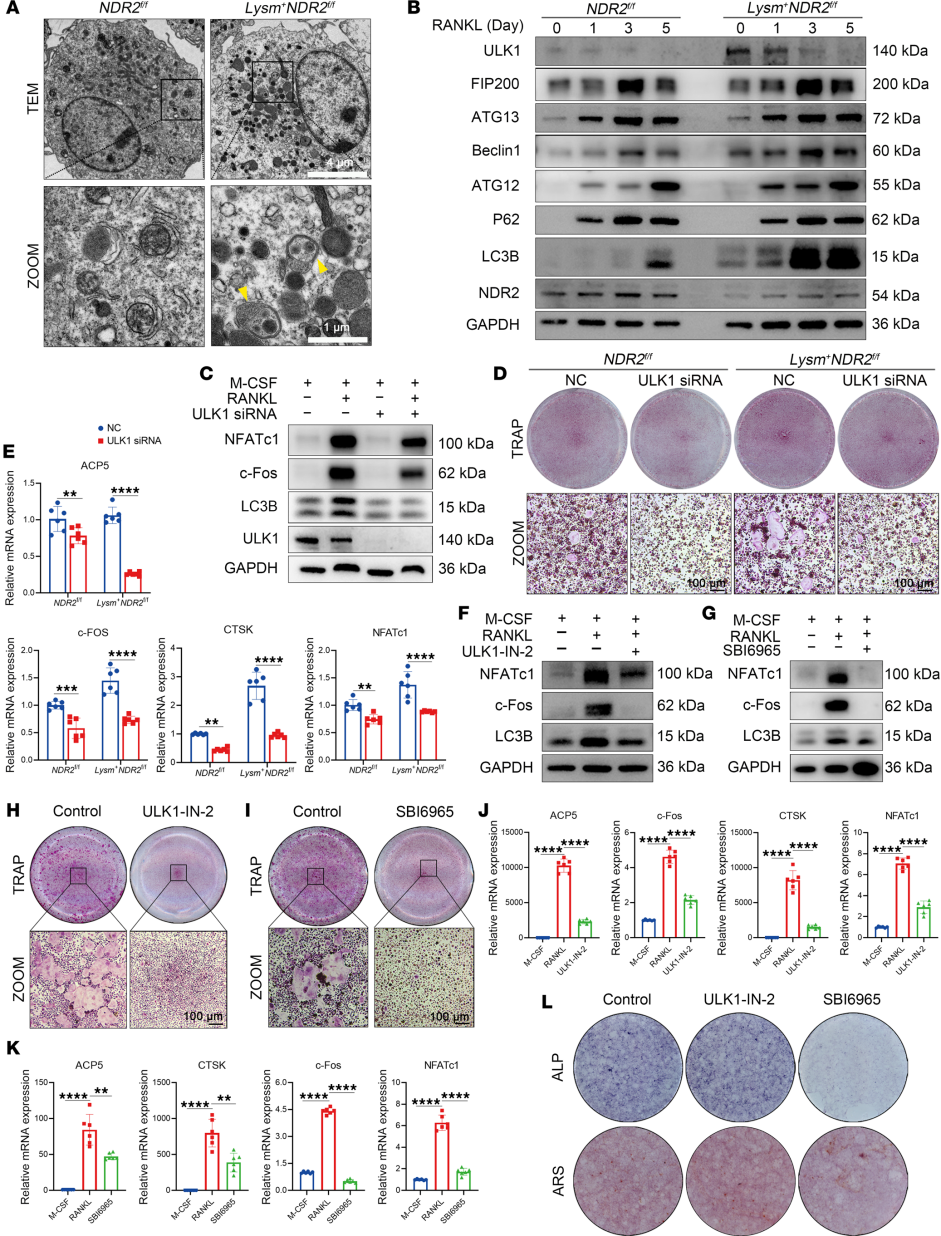
NDR2 regulated osteoclastogenesis via ULK1. (**A**) Transmission electron microscope (TEM) image of osteoclastic progenitor cells. The image below is magnified. The yellow arrowheads mark the autophagosome. RANKL (25 ng/mL) was administered to stimulate the cells for 48 hours. (**B**) Detection of autophagy-associated marker proteins in *Lysm^–^NDR2^fl/fl^* and *Lysm^+^NDR2^fl/fl^* mouse-derived bone marrow macrophages (BMMs) at different time points induced by osteoclasts. (**C**) ULK1 siRNA was transfected into BMMs with *Lysm^+^NDR2^fl/fl^* genotype, followed by stimulation with RANKL after 12 hours. Protein extraction was performed 48 hours later for Western blot. (**D**) TRAP staining images of BMMs derived from *Lysm^–^NDR2^fl/fl^* and *Lysm^+^NDR2^fl/fl^* after stimulation of RANKL. Enlarge image below. (**E**) The differential expression of osteoclast-related genes was assessed by qPCR following ULK1 siRNA treatment (*n* = 6). (**F** and **G**) The levels of osteoclast-related proteins and autophagy were altered following the addition of ULK1 inhibitors, namely ULK1-IN2 (200 nmol) and SBI6965 (20 nmol). (**H** and **I**) TRAP staining images with or without ULK1 inhibitors. (**J** and **K**) Osteoclast-related gene changes were statistically analyzed with or without ULK1 inhibitors. (**L**) The osteogenic differentiation of the MC3T3-E1 cell line was induced for 21 days, followed by alizarin red staining and induced for 14 days with alkaline phosphatase staining. Statistical analyses were determined by 1-way ANOVA (**J** and **K**), and 2-way ANOVA (**E**). **P < 0.01, ***P < 0.001, and ****P < 0.0001. Data were presented as mean ± SD.

Autophagy plays a pivotal role in osteoclastogenesis; the inhibition of autophagy was found to impede OC differentiation, consistent with previous research (Supplemental Figure 4, B and C). We investigated whether NDR2 knockdown could modulate autophagy in OCs, and we subsequently assessed the level of autophagy in proosteoclast cells. The *Lysm^+^NDR2^fl/fl^* group exhibited an increased number of autophagosomes in proosteoclast cells (Figure 4A), indicating a significant upregulation of autophagy in this group. Furthermore, the upregulation of LC3, an essential autophagy marker, following NDR2 KO suggests the induction of active autophagy (Figure 4B). ULK1 (UNC-51–like kinase 1), a core component of the ULK1 complex, plays a crucial role in initiating autophagy (30). After NDR2 knockdown, the expression levels of ULK1 were elevated compared with those in the *NDR2^fl/fl^* group, whereas they were downregulated upon NDR2 overexpression (Supplemental Figure 4E). Therefore, we postulated that NDR2 modulates autophagy by influencing ULK1, thereby exerting regulatory control over osteoclastogenesis. The alterations observed in other autophagy markers may be attributed to variations in ULK1 levels.

ULK1 siRNA was employed to further investigate whether NDR2 knockdown promotes osteoclastogenesis through ULK1. Knockdown of ULK1 resulted in reduced levels of the autophagy marker LC3B and inhibition of OC-related markers (Figure 4C). TRAP staining demonstrated that the OC-promoting effect of NDR2 knockdown could be rescued by ULK1 siRNA (Figure 4D). Consistent with the protein results, downregulation of OC-related gene expression was observed upon treatment with ULK1 siRNA (Figure 4E and Supplemental Figure 4D). An ULK1-specific inhibitor (ULK1-IN2) was also utilized to confirm that NDR2 knockdown–induced overactivity in osteoclastogenesis is mediated by ULK1, which significantly decreased autophagy levels and inhibited OC differentiation (Figure 4, F, H, and J). Similarly, SBI6965, another selective inhibitor for ULK1, exhibited a similar inhibitory effect on autophagy and OC differentiation (Figure 4, G, I, and K). Based on these findings, we concluded that upregulation of ULK1 induced by NDR2 knockdown enhanced osteoclastogenesis via promotion of autophagy. Simultaneously, ALP and ARS staining were performed to validate the effect of these 2 inhibitors on osteoblastogenesis. The results demonstrate that SBI6965 exhibited an inhibitory effect on osteoclastogenesis at the concentration that effectively suppressed OB differentiation, whereas ULK1-IN2 did not exhibit such an effect (Figure 4L).

### NDR2 KO enhanced ULK1 stabilization and promoted mitophagy.

**Figure 5:**
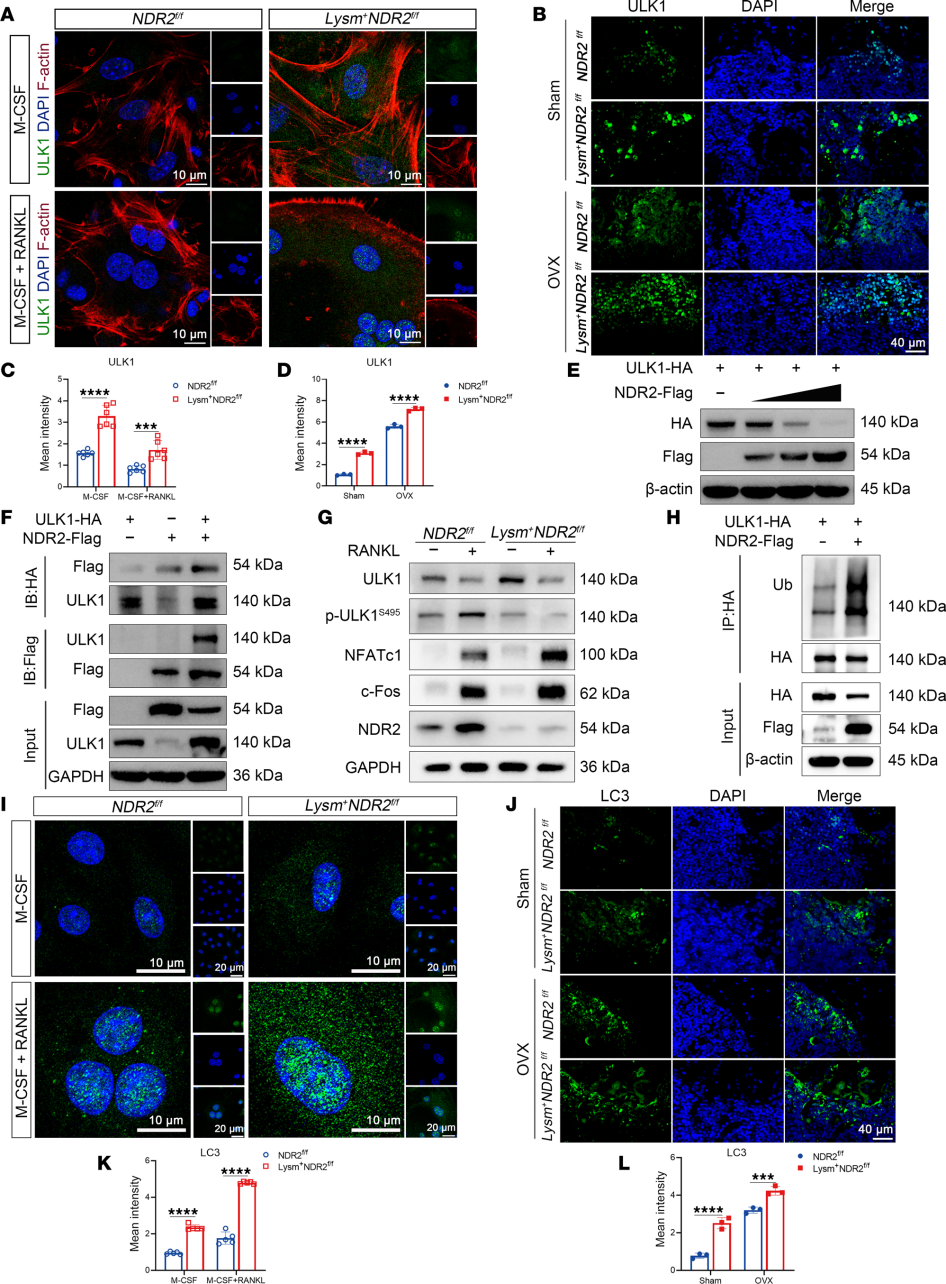
NDR2 KO enhanced ULK1 stabilization. (**A** and **C**) Cellular immunofluorescence staining and statistical analysis of ULK1. RANKL (25 ng/mL, 5 days), ULK1 (green), F-actin (red), and DAPI (blue). (**B** and **D**) Fluorescence staining and statistical analysis of tissue sections for ULK1. ULK1 (green) and DAPI (blue). (**E**) The HeLa cell lines were subjected to overexpression of ULK1-HA plasmid and subsequent transfection with NDR2-Flag plasmid in a concentration gradient, followed by Western blot. (**F**) The HeLa cell lines were transfected with ULK1-HA and NDR2-Flag plasmids, followed by the detection of the interaction between ULK1 and NDR2 proteins through Co-IP. (**G**) The Western blot of *Lysm^–^NDR2^fl/fl^* and *Lysm^+^NDR2^fl/fl^* BMMs were analyzed. (**H**) The HeLa cell lines were transfected with ULK1-HA and NDR2-Flag plasmids, followed by the detection of the ubiquitination of ULK1 through Co-IP. (**I** and **K**) Immunofluorescence staining and statistical analysis (*n* = 6) of LC3 was performed on cellular samples, followed by. RANKL (25 ng/mL, 2 days), LC3 (green), and DAPI (blue). (**J** and **L**) Fluorescence staining and statistical analysis were performed on tissue sections to assess LC3 expression (*n* = 3). ULK1 (green) and DAPI (blue). Statistical analyses were determined by 2-way ANOVA (**C**, **D**, **K**, and **L**). ***P < 0.001 and ****P < 0.0001. Data were presented as mean ± SD.

To explore how NDR2 regulates ULK1 to affect osteoclastogenesis, we initially conducted fluorescence staining for ULK1 and observed an enhanced fluorescence intensity following NDR2 knockdown (Figure 5, A and C). During OC differentiation, autophagy levels progressively increased, leading to the degradation of ULK1 along with autophagosomes in lysosomes. Consequently, the level of ULK1 decreased in the late stage compared with the early stage. Subsequently, tissue section staining confirmed higher expression of ULK1 in the *Lysm^+^NDR2^fl/fl^* group compared with the *NDR2^fl/fl^* group, with further enhancement of ULK1 fluorescence intensity after OVX and aging modeling (Figure 5, B and D, and Supplemental Figure 5, A and B). Additionally, gradient overexpression of NDR2 in HeLa cells resulted in reduced levels of ULK1 protein (Figure 5E), and coimmunoprecipitation (Co-IP) analysis validated the interaction between NDR2 and ULK1 proteins (Figure 5F).

ULK1 can undergo phosphorylation by mTORC1 (31), AMPK (30), and p38 MAPK–related kinases (24). AMPK phosphorylates ULK1 at Ser317, Ser777, Ser467, Ser555, Thr574, and Ser637 to enhance its activity and initiate autophagy. Conversely, mTORC1 and p38 MAPK phosphorylate ULK1 at Ser504 and Ser757 to inhibit autophagy. In breast carcinogenesis, NDR2 phosphorylates ULK1 at Ser495, leading to its ubiquitination and subsequent degradation, thereby exerting inhibitory effects on autophagy (26). Our previous findings demonstrate the substantial increase in ULK1 levels in NDR2^–/–^ OCs (Figure 4B). Based on these observations, we hypothesized that the kinase NDR2 might be responsible for phosphorylating ULK1 at Ser495. To test this hypothesis, we assessed the phosphorylation status of ULK1 at Ser495. As depicted in Figure 5G, knockdown of NDR2 resulted in elevated ULK1 levels compared with the *NDR2^fl/fl^* group; however, it significantly inhibited the phosphorylation of ULK1 at Ser495. To demonstrate that NDR2 regulates OC differentiation through the phosphorylation of the ULK1 Serine 495 site, we mutated ULK1 Serine 495 to Alanine (ULK1^S495A^). Using lentiviral vectors, we overexpressed both ULK1^WT^ and ULK1^S495A^ in BMMs. Western blot and TRAP staining results (Supplemental Figure 4, F and G) reveal that ULK1^S495A^ significantly enhanced osteoclastogenesis compared with overexpression of NDR2^WT^. Therefore, NDR2 orchestrated osteoclastogenesis by phosphorylating ULK1 at Ser495 and facilitating its subsequent degradation by ubiquitination (Figure 5H). ULK1 forms the complex with FIP200, ATG13, and ATG101, which activates the class III phosphatidylinositol 3-kinase (PIK3C3) complex and initiates the process of autophagy (32). Subsequently, we investigated whether the NDR2/ULK1 axis regulates autophagy levels. Immunofluorescence staining of both cells and tissues revealed an upregulation in LC3 levels following NDR2 knockdown, indicating enhanced autophagy induction (Figure 5, I–L).

**Figure 6:**
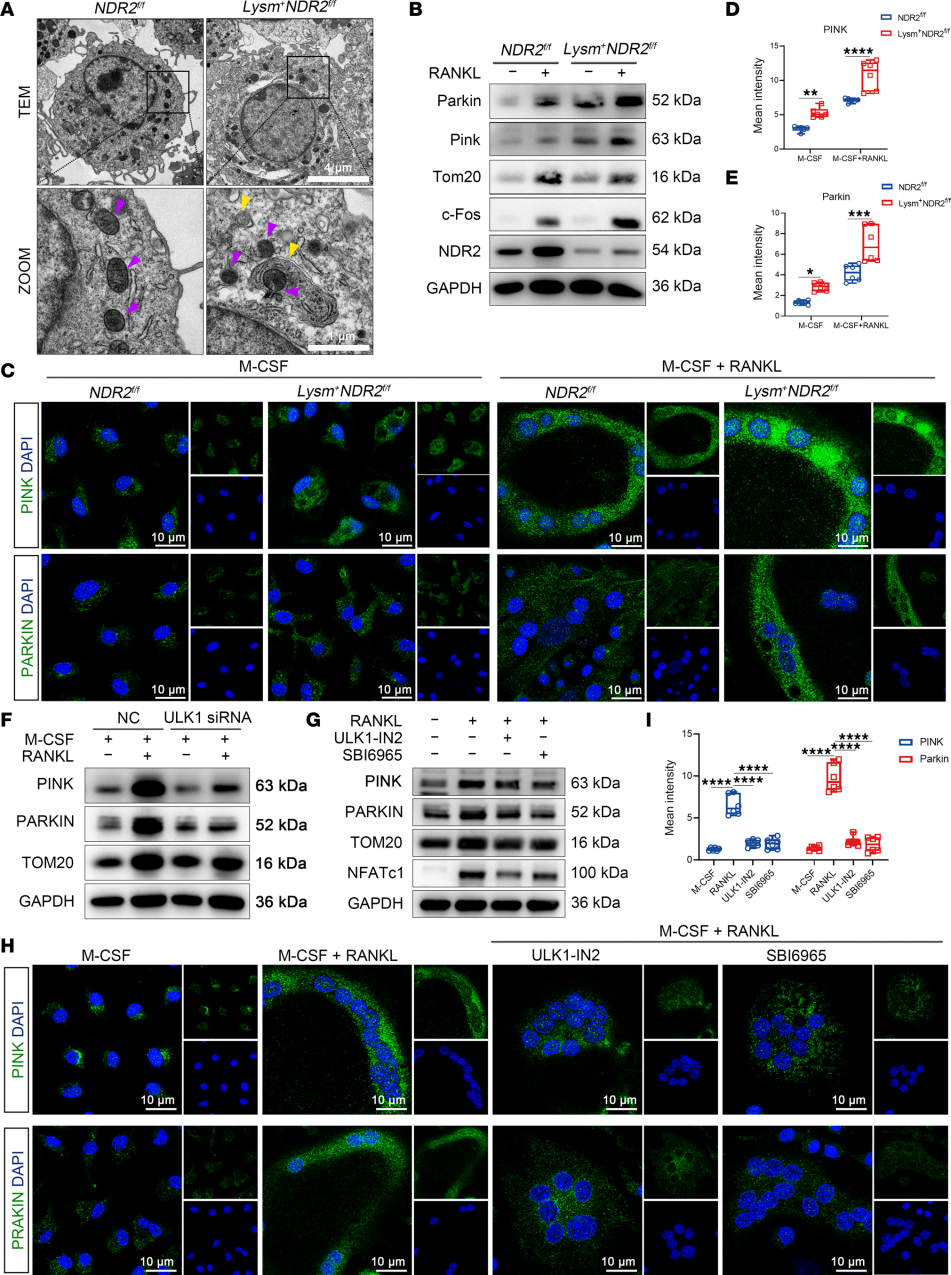
NDR2 KO promoted mitophagy via ULK1. (**A**) Representative image of the transmission electron microscope (TEM), with purple arrows indicating mitochondria and yellow arrows indicating autophagosomes. (**B**) Western blot was used to detect the classical regulatory signaling pathway of mitophagy. Tom, mitochondrial outer membrane protein. (**C**) Immunofluorescence staining images of *Lysm^+^NDR2^fl/fl^* BMMs were obtained to visualize the expression of PINK and PARKIN. The staining was performed using antibodies against PINK/PARKIN (green) and DAPI for nuclear counterstaining (blue). (**D** and **E**) Quantitative analysis was conducted to determine the fluorescence intensities of PINK and PARKIN (*n* = 6). (**F** and **G**) BMMs derived from *Lysm^+^NDR2^fl/fl^* mice were transfected with ULK1 siRNA (**F**) or treated with ULK1 inhibitor (**G**), and the classical mitophagy signaling pathway was analyzed by Western blot. (**H**) PINK and PARKIN immunofluorescence staining images of BMMs derived from *Lysm^+^NDR2^fl/fl^* mice treated with ULK1 inhibitor. PINK/PARKIN (green) and DAPI (blue). (**I**) Statistical analysis of PINK and PARKIN fluorescence intensity (*n* = 6). Statistical analyses were determined by 1-way ANOVA (**I**) and 2-way ANOVA (**D** and **E**). *P < 0.05, **P < 0.01, ***P < 0.001, and ****P < 0.0001. Data were presented as mean ± SD.

Mitophagy is employed to eliminate redundant or dysfunctional mitochondria in order to maintain the equilibrium of mitochondrial number and mass, thereby enabling cells to sustain normal physiological function even under conditions of malnutrition or external stimulation (33). ULK1 activation promotes the formation of autophagosomes and facilitates damaged mitochondrial clearance (34). To investigate the potential effect of NDR2 KO on mitophagy, we assessed relevant indicators associated with mitophagy. Compared with the *NDR2^fl/fl^* group, transmission electron microscopy (TEM) revealed a higher abundance of autophagosomes containing encapsulated mitochondria in *Lysm^+^NDR2^fl/fl^* group (Figure 6A). Knockdown of NDR2 resulted in a reduction of TOM20, a mitochondrial membrane protein. PTEN-induced kinase 1 (PINK) serves as a sensor for mitochondrial damage and initiates mitophagy signaling. PARKIN facilitates the ubiquitination of mitochondrial substrates, leading to the accumulation of receptor proteins such as P62 on the outer mitochondrial membrane. This accumulation subsequently recruits ubiquitinated products to autophagosomes through binding with LC3. PINK and PARKIN expression levels were significantly upregulated in the NDR2-KO group, indicating a higher degree of mitophagy compared with the *NDR2^fl/fl^* group (Figure 6, B–E). The knockdown of ULK1 effectively suppressed the upregulation of mitophagy levels induced by NDR2 knockdown (Figure 6F), and a similar effect was observed upon inhibition of ULK1, resulting in reduced fluorescence intensity of PINK and PARKIN as well as inhibition of OC formation (Figure 6, G–I). Collectively, knockdown of NDR2 impeded the degradation of ULK1 while enhancing autophagy and mitophagy.

### ULK1-IN2 rescued bone mass loss in both OVX model and Lysm^+^NDR2^fl/fl^ mice.

**Figure 7:**
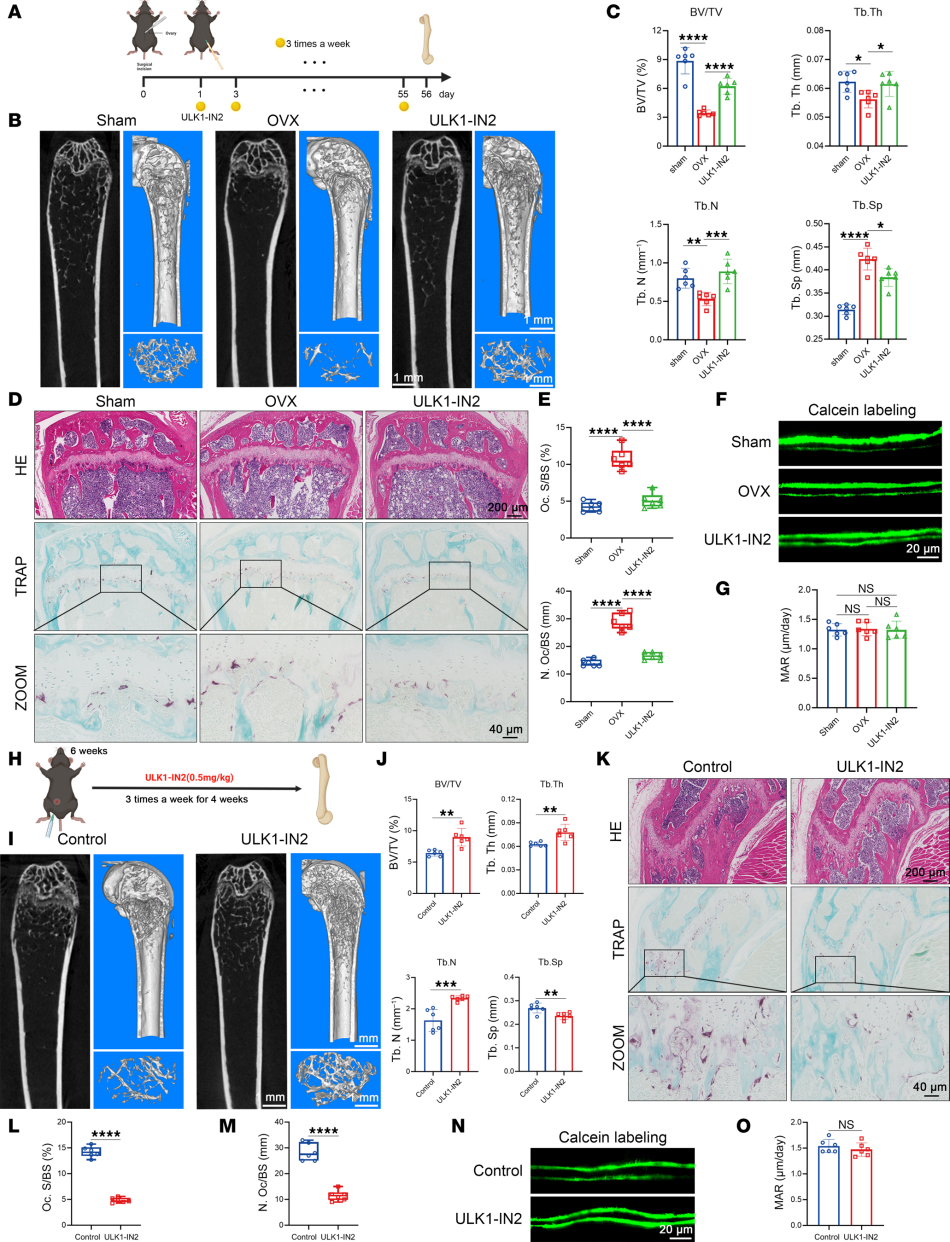
ULK1-IN2 rescued bone mass loss in both OVX model and *Lysm^+^NDR2^fl/fl^* mice. (**A**) Schematic diagram of the model. Eight-week-old WT female mice underwent ovariectomy (OVX), followed by i.p. injection of ULK1-IN2 (0.5 mg/kg) on the second day after surgery, administered 3 times per week for a duration of 8 weeks. On day 56, the mice were euthanized to obtain tissue samples for subsequent experiments. (**B**) Representative 2D and 3D μCT images of the femur were obtained. (**C**) Statistical analysis was conducted on bone mass parameters of the femur (*n* = 6). (**D**) Histological staining using H&E and TRAP staining was performed on sections of the femur. (**E**) Histomorphological analysis of TRAP staining (*n* = 6). (**F**) Double-labeled calcein fluorescence image. Mice were i.p. injected with calcein staining solution on days 3 and 10 before sacrifice. (**G**) Statistical analysis of mineralization rate (*n* = 6). (**H**) Schematic diagram of the model. Six-week-old *Lysm^+^NDR2^fl/fl^* mice were i.p. administered ULK1-IN2 (0.5 mg/kg) 3 times per week for a duration of 4 weeks, following which the mice were euthanized to obtain tissue samples for subsequent experimental analysis. (**I**) Representative 2D and 3D images of femur. (**J**) Statistical analysis of femoral bone mass parameters (*n* = 6). (**K**) H&E staining and TRAP staining of femur sections. (**L** and **M**) Histomorphological analysis of TRAP staining (*n* = 6). (**N**) Double-labeled calcein fluorescence image. (**O**) Statistical analysis of mineralization rate (*n* = 6). Statistical analyses were determined by 2-tailed Student’s *t* test (**J**, **L**, and **M**), and 1-way ANOVA (**C**, **E**, and **G**). *P < 0.05, **P < 0.01, ***P < 0.001, and ****P < 0.0001. Data were presented as mean ± SD.

Considering the inhibitory effect of SBI6965 on osteoblastogenesis (Figure 4L), ULK1-IN2 was chosen for subsequent in vivo experiments. WT mice were subjected to OVX modeling followed by continuous administration of the ULK1 inhibitor ULK1-IN2 throughout the modeling process, and tissue samples were collected after 2 months (Figure 7A). After OVX modeling, estrogen decreased and body weight of mice was upregulated, and ULK1-IN2 showed slight inhibition of weight gain (Supplemental Figure 6A). ULK1-IN2 administration partially ameliorated the osteoporotic bone loss induced by OVX, as evidenced by the upregulation of BV/TV (%) and Tb.Th (mm^–1^), along with the downregulation of Tb.Sp (mm) (Figure 7, B and C, and Supplemental Figure 6, B and C). ULK1 inhibitor partially reversed the hyperactivity of osteoclastogenesis induced by OVX, as shown by TRAP staining (Figure 7D). Histomorphological analysis revealed inhibitory effects on both Oc.S/BS and Oc.N/BS (Figure 7E). The calcein staining results also indicate that there were no significant differences in bone formation following ULK1-IN2 treatment (Figure 7, F and G). We also examined the number of OBs by OCN staining, which suggested that there was no significant difference in the number of OBs among the groups (Supplemental Figure 6, G and H).

To investigate the potential of ULK1 inhibitors in enhancing bone mass in *Lysm^+^NDR2^fl/fl^* mice, 6-week-old *Lysm^+^NDR2^fl/fl^* mice were administered ULK1 inhibitors via i.p. injection for a duration of 4 weeks (Figure 7H), while the body weight of the mice remained unchanged (Supplemental Figure 6D). As shown in 2D and 3D images, the number of bone trabeculae increased, and bone mass was effectively enhanced (Figure 7, I and J, and Supplemental Figure 6, E and F). The ULK1 inhibitor treatment group demonstrated a decrease in the number of TARP^+^ OCs (Figure 7, K–M), implying that inhibition of ULK1 could alleviate the enhancement of osteoclastogenesis induced by NDR2 knockdown. In line with the WT mouse OVX model, ULK1-IN2 demonstrated inhibition of in vivo osteoclastic activity, while its effect on osteogenic activity was minimal (Figure 7, N and O, and Supplemental Figure 6, I and J).

Mitochondrial autophagy indicators PINK and PARKIN in myeloid cells were upregulated after OVX modeling, and ULK1-IN2 treatment inhibited this trend (Supplemental Figure 7, A–C). Similarly, *Lysm^+^NDR2^fl/fl^* mice showed a reduction in mitophagy after ULK1 inhibitors (Supplemental Figure 7, D–F). Overall, the administration of ULK1 inhibitor effectively rescued bone mass loss in both the OVX model WT mice and *Lysm^+^NDR2^fl/fl^* mice.

### An inverse correlation between NDR2 and the prevalence of osteoporosis.

**Figure 8:**
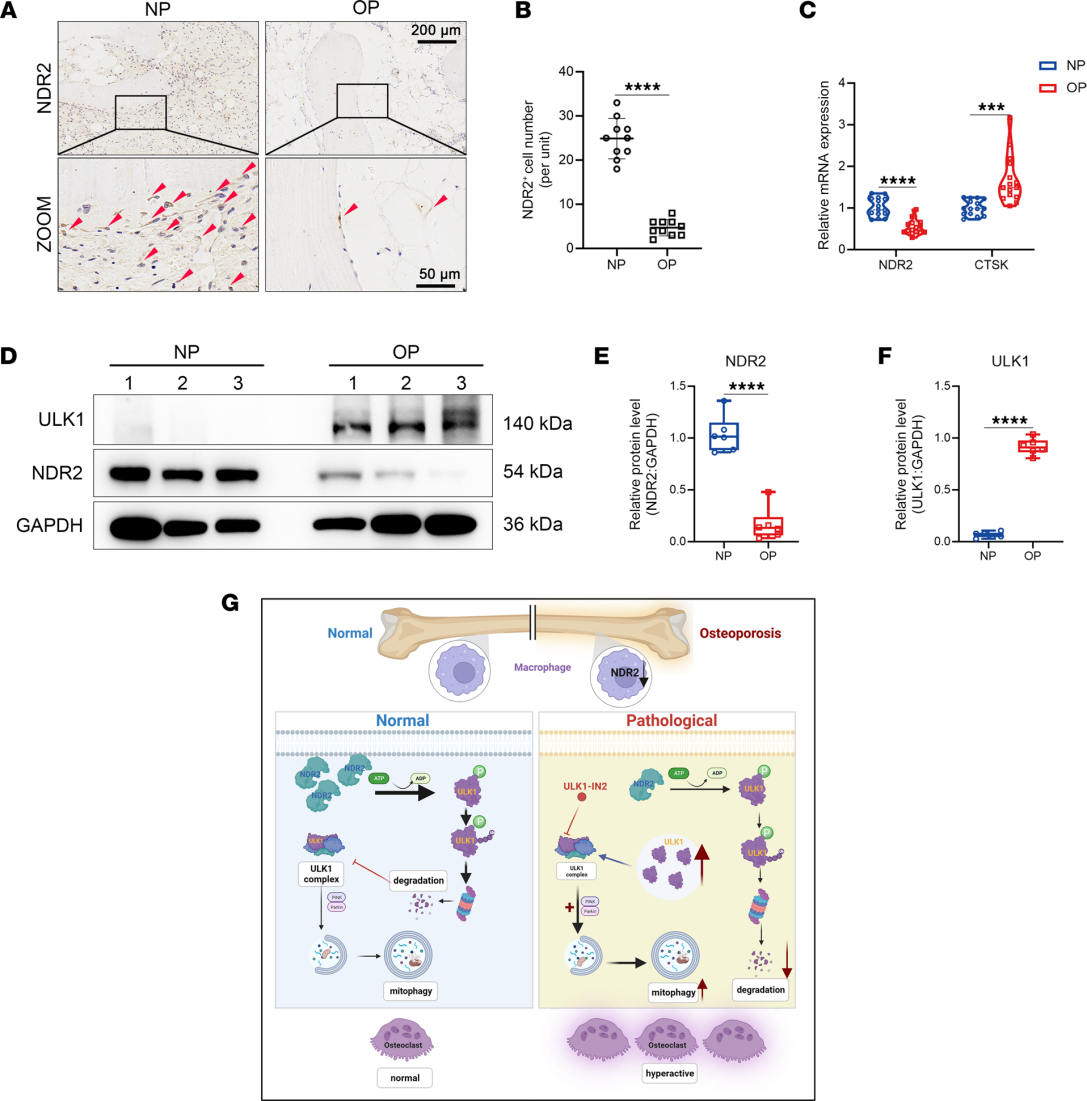
An inverse correlation between NDR2 and osteoporosis. (**A**) Representative images of IHC staining of surgically removed cancellous bone specimens from normal bone mass and osteoporosis patients. Red arrows mark NDR2^+^ cells. (**B**) Statistical analysis of NDR2^+^ cells (*n* = 10). (**C**) The expression levels of *NDR2* and *CTSK* (genes associated with osteoclast activity) in peripheral blood mononuclear cells derived from patients were quantified using qPCR (*n* = 15). (**D**) The protein levels of NDR2 and ULK1 in peripheral blood mononuclear cells from patients were assessed using Western blot. (**E** and **F**) Statistical analysis of the relative expression of NDR2 and ULK1 proteins (*n* = 6). (**G**) Schematic illustration of the effect of NDR2 on osteoclastogenesis (created with BioRender.com). In the physiological state, ULK1 functions as a pivotal component of the ULK1 complex and initiates mitophagy. NDR2 regulates osteoclast differentiation through phosphorylation of ULK1 at Ser495, thereby facilitating its coupled ubiquitination and degradation, thus restricting the extent of mitophagy. In osteoporosis, there is a reduction in the expression level of NDR2, leading to weakened phosphorylation of ULK1^S495^ and subsequent cytoplasmic accumulation of ULK1. The intracellular buildup of ULK1 exacerbates mitophagy levels and promotes excessive osteoclast differentiation. Statistical analyses were determined by 2-tailed Student’s *t* test (**B**, **C**, **E**, and **F**). ***P < 0.001 and ****P < 0.0001. Data were presented as mean ± SD.

The association between NDR2 and osteoporosis incidence was investigated by analyzing cancellous bone samples that were surgically removed from patients. IHC staining revealed a lower number of NDR2^+^ cells in osteoporosis group compared with the normal bone density group (Figure 8, A and B). In vitro verification using peripheral blood mononuclear cells showed that gene expression of *NDR2* was significantly downregulated in osteoporotic patients, contrary to the trend observed for osteoclastic marker *CTSK* (Figure 8C). Western blotting indicated lower levels of NDR2 protein in human peripheral mononuclear cells from the osteoporosis group (Figure 8, D and E), suggesting a negative correlation between NDR2 level and osteoporosis incidence. ULK1 levels were found to be upregulated in osteoporotic patients as a downstream regulatory molecule of NDR2, indicating higher autophagy levels than controls (Figure 8, D and F). Based on these findings, it is suggested that the NDR2/ULK1/mitophagy axis may play an important role in the occurrence and development of osteoporosis (Figure 8G), providing insights for clinical prevention and treatment.

## Discussion

Osteoporosis, a prevalent bone metabolic disorder, primarily arises from the dysregulation between OB-mediated bone formation and OC-driven bone resorption (27). Factors such as postmenopausal estrogen decline, aging, and chronic contribute to the development of osteoporosis by predominantly promoting excessive activity of OCs (35–38). However, the precise underlying mechanism remains incompletely elucidated.

Mitochondria serve as the primary energy generators within cells (39). By means of mitophagy, cells are able to regulate both the quantity and quality of mitochondria, thereby preventing the release of detrimental substances from damaged mitochondria while meeting the diverse energy demands associated with various cellular activities (40–42). Furthermore, it has been demonstrated that mitophagy is closely intertwined with bone metabolism (42–47). Through this process, damaged mitochondria can be eliminated to safeguard OBs against apoptosis (48). Recent investigations have revealed that mitophagy promotes OC proliferation, whereas inhibition of such autophagy induces OC apoptosis (49). Epigallocatechin-3-gallate (EGCG) modulates mitophagy to suppress OC differentiation through the AKT and MAPK signaling pathways (50). As a crucial constituent of the initiation complex for autophagy, ULK1 governs either the initiation of general autophagy or specifically targets mitochondrial degradation (21, 51–53), hence compromising ULK1 function weakens mitophagy.

NDR2, a member of the Hippo signaling family, functions as a kinase and phosphorylates downstream proteins involved in signal transduction. Specifically, NDR2 phosphorylates YAP/TAZ, leading to its degradation and subsequent inhibition of the Hippo signaling pathway (54, 55). TAZ is a key regulator of osteoclastogenesis and osteoporosis. Our and others’ previous studies have demonstrated that KO of TAZ promotes osteoclastogenesis, whereas TAZ overexpression suppresses RANKL-induced OCs formation (56–58). However, contrary to our expectations, NDR2 KO significantly promotes osteoclastogenesis, which implies that alternative regulatory mechanisms bypass YAP/TAZ signaling. We then evaluated the effects of NDR2 KO on autophagy for the vital role of autophagy in OC differentiation. Notably, upon NDR2 KO, there was an inhibition in ULK1 phosphorylation degradation that subsequently led to increased mitophagy levels promoting OC differentiation. Surprisingly, reduced expression levels of NDR2 were also observed in mouse models with OVX or aging as well as patients diagnosed with osteoporosis.

This study holds immense significance in further elucidating the role of kinase phosphorylation and ULK1-mediated autophagy in osteoporosis-related disorders, thereby offering potentially novel targets and a theoretical foundation for the prevention and treatment of bone metabolic diseases. The present study exhibits certain limitations that warrant further investigation. The expression level of NDR2 is upregulated during OC differentiation, exerting a negative regulatory effect on this process and preventing excessive activation. However, in both the osteoclastogenesis model and patient samples, there was a decrease in NDR2 expression accompanied by reduced ability to inhibit OC differentiation. The precise mechanism underlying the downregulation of NDR2 expression in macrophages induced by osteoporosis remains elusive. Additionally, there is a paucity of research on the role of mitophagy in regulating OC differentiation, necessitating further exploration into its potential mechanisms.

In conclusion, our study has unveiled a potentially novel regulatory mechanism of OC differentiation mediated by the NDR2/ULK1/mitophagy axis, which has been validated in mice pathological models and osteoporosis patients. These findings offer innovative strategies for clinical prevention and treatment of osteoporosis.

## Methods

### Sex as a biological variable.

Our study examined both male and female mice and humans.

### Reagents and antibodies.

MC3T3 cell line was obtained from American Type Culture Collection (ATCC, TIB-71). RANKL and M-CSF were obtained from R&D Systems. ULK1-IN2 (HY-143466), SBI6965 (HY-16966), and Chloroquine (HY-17589A) were obtained from MedChemExpress. Antibodies of FIP200 (HA601107), ATG12 (M1701-4), and TOM20 (ET1609-25) were purchased from HUABIO. Antibodies of TRAP/ACP5 (ab191406) and RUNX2 (ab133261) were purchased from Abcam. Antibodies of P65 (8242), p-P65 (3033), P38 (9212), p-P38 (4511), JNK (9252), p-JNK (9255), ERK (5013), p-ERK (4370), FLAG (14793S), ULK1 (8054S), ATG13 (13468S), BECLIN1 (3495T), P62 (5114), LC3 (3868), YAP (14074), p-YAP (13008), TAZ (83669), and c-FOS (31254) were purchased from Cell Signaling Technology. Antibodies of GAPDH (60004-1-Ig), PINK (23274-1-AP), PARKIN (14060-1-AP), and OCN (23418-1-AP) were purchased from Proteintech. Antibodies of NFATc1 (sc-7294) and HA (sc-7392) were purchased from Santa Cruz Biotechnology Inc. Antibodies of NDR2 (TA505176) were purchased from Origene. Antibodies of p-ULK1^S495^ were provided from Chen Song lab (Henan Provincial People’s Hospital, Academy of Medical Sciences, Zhengzhou University). DAPI (C0065) was a product of Solarbio. Goat anti–mouse HRP (FDM007) and goat anti–rabbit HRP (FDR007) were purchased from Fudebio-tech. Anti–rabbit IgG Fab2 Alexa Fluor 488 conjugate antibody and anti–mouse IgG Fab2 Alexa Fluor 555 conjugate antibody were purchased from Cell Signaling Technology (4412S and 4409S, respectively).

### Mice.

*NDR2^fl/fl^* mice, a C57BL/6J background, were gifts from B. Hemmings (Friedrich Miescher Institute for Biomedical Research). *Lysm-Cre* mice C57BL/6J were provided by Gempharmatech Co. Ltd. In all experiments, *Lysm^+^NDR2^fl/fl^* mice were compared with their WT littermates (*NDR2^fl/fl^*).

### Human tissue from normal bone mass and osteoporosis patients.

Informed consent was obtained from each patient. Surgically removed cancellous bone specimens from normal bone mass and osteoporosis patients were collected from patients. Peripheral blood samples were obtained from the patients, and monocytes were isolated using the human monocyte extraction kit (Solarbio, P9011) for subsequent protein and mRNA extraction.

### Cell culture, OC, and OB differentiation.

Femur and tibia bone marrow from 8-week-old C57BL/6J mice were extracted using a syringe, followed by culturing in α-minimum essential medium (α-MEM) supplemented with 10 % FBS (Thermo Fisher Scientific), 1 % penicillin-streptomycin, and 50 ng/mL M-CSF (Amizona Scientific). The cultures were maintained in a humidified atmosphere of 95% air and 5% CO_2_ at 37°C with medium renewal every 2 days until reaching preosteoclast cells at 90% confluency. BMMs were then differentiated into mature OCs through stimulation with 50 ng/mL M-CSF and 25 ng/mL RANKL (R&D Systems), subsequently utilized for experiments such as Western blot, quantitative PCR (qPCR), and TRAP staining.

The MC3T3 cell line was induced by supplementing the culture medium with 5 μM ascorbic acid and 1 mM β-glycerophosphate. ALP staining was performed on day 14, followed by ARS staining on day 21. The culture medium was refreshed every other day until sample collection. The cells were cultured in a humidified atmosphere of 95% air and 5% CO_2_ at 37°C.

### Western blot.

Cells were seeded at a density of 1 × 10^6^ per well in 6-well plates and treated with ULK1 inhibitors after 48 hours. Total protein was extracted from adherent cells using RIPA lysate, followed by centrifugation at 12,000*g* for 15 minutes to collect the supernatant. The proteins were separated via 10% SDS-PAGE and transferred onto PVDF membranes. Following a 1-hour blocking step with 5% skim milk powder at room temperature, the cells were subjected to overnight incubation with primary antibody at 4°C and subsequent incubation with secondary antibody for 1 hour on a shaker. Protein strips were then exposed to ECL solution using a LAS-4000 Science Imaging System (Fujifilm), and acquired images were subsequently analyzed and processed using ImageJ (NIH).

### qPCR.

Cells were cultured at a density of 5 × 10^5^ per well in 12-well plates with or without ULK1 inhibitors treatment. Total RNA was extracted using the TRIzol reagent extraction protocol, followed by reverse transcription into cDNA using the HiFiScript cDNA Synthesis Kit (CW2569, CWBIO). The qPCR amplification reaction was performed utilizing Hieff qPCR SYBR Green Master Mix (11201ES08, Yeasen). The mRNA expression levels of the target genes were quantified by calculating Ct values and normalized to GAPDH levels. Primer sequences are listed in Table 1 and Table 2.

### Immunofluorescence staining.

BMMs were seeded at a density of 1 × 10^5^ cells per well on slides precoated in a 24-well plate and cultured in α-MEM supplemented with 50 ng/mL M-CSF for 24 hours prior to subsequent experiments. The medium was aspirated and the cells were fixed with 4% paraformaldehyde (G1101, Servicebio) for 15–20 minutes after being washed 3 times with PBS. Triton X-100 (0.1%) for 15 minutes at room temperature and QuickBlock immunostaining blocking solution (P0260, Beyotime) was then applied at room temperature for 1 hour. The blocking solution was discarded and the corresponding primary antibody was added for overnight incubation at 4°C. The plates were washed thrice with PBS, each time for 10 minutes, followed by incubation with fluorescent secondary antibody and DAPI in a dark room at room temperature for 1 hour. Finally, the slides were sealed using an antiquenching agent. Fluorescence images were captured using Nikon A1 Ti confocal microscope and analyzed through ImageJ (NIH).

Following dewaxing of the tissue sections, antigen retrieval was performed by overnight soaking in sodium citrate buffer at 55°C. After slowly cooling to room temperature, QuickBlock immunostaining sealer was applied and allowed to cure for 1 hour at room temperature before addition of primary antibody and overnight incubation at 4°C. The slides were subjected to 3 washes with TBST prior to incubation with fluorescent secondary antibody and DAPI for 1 hour at room temperature, followed by another 3 washes with TBST. The antiquenching agent was hermetically sealed before fluorescence scanning.

### IHC staining.

For IHC staining, sections were submerged with sodium citrate at 55°C overnight for antigen retrieving. After being blocked with BSA for 1 hour, the sections were incubated with anti-NDR2 antibody at 4°C overnight and washed with PBS for 3 times and incubated with anti-mouse secondary antibody (ZsBio). Sections were washed with PBS for 3 times and stained with DAB solution (ServiceBio) at RT for 5–10 minutes. The images were captured by microscope (Nikon).

### TRAP staining.

After fixation with 4% paraformaldehyde as recommended by the manufacturer, cells were stained for TRAP using a leukocyte diagnostic kit (Cosmo Bio). Duplicate wells of 24-well plates revealed the presence of multinucleated OCs (>3 nuclei) and mononucleated TRAP^+^ cells.

### Transfection of NDR2 and ULK1 siRNA.

siRNAs targeting NDR2 and ULK1 were purchased from RiboBio Co. Ltd. At day 0, BMMs were transfected and subsequently cultured in 12-well plates (5 × 10^5^ cells per well) with RANKL stimulation for 0, 1, 3, and 5 days. Lipofectamine RNAiMAX (13778150, Invitrogen) was utilized for transfection in accordance with the manufacturer’s instructions. The efficacy of knockdown was assessed on day 5 after RANKL treatment via Western blot and qPCR, as well as TRAP staining as previously mentioned.

### ALP and ARS staining.

For the in vitro differentiation of OBs, MC3T3-E1 cells were cultured in α-MEM supplemented with 10% FBS containing 1 mM β-glycerophosphate and 5 μM L-ascorbic acid 2-phosphate. The culture medium was refreshed every 2 days. After a 14-day incubation period, ALP staining was conducted to determine the number of positive cells. To investigate the effect of ULK1 inhibitors on mineralization, MC3T3-E1 cells were seeded at a density of 1 × 10^4^ cells/well in 24-well plates. After being treated with osteogenic medium containing ULK1-IN2 and SBI6965 for 21 days, the cells were gently washed twice with PBS, fixed in 4% paraformaldehyde for 15–20 minutes, and stained with Alizarin Red S solution (Cyagen Biosciences) at 37°C for 5 minutes to visualize extracellular matrix mineralization nodules. The images were captured using an inverted microscope equipped with a digital camera.

### μCT scanning and analysis.

Mice were euthanized to obtain skull, femur, and tibia tissues. The bone tissues were subsequently fixed in 4% paraformaldehyde for 48 hours before the femur and tibia tissues underwent μCT scanning (SkyScan1275, Bruker) with the following parameters: a source voltage of 50 kV, a source current of 450 μA, an aluminum (AI) filter thickness of 0.5 mm, a pixel size of 9 μm, and rotation step of 0.4°. Furthermore, the acquired images were reconstructed using NRecon software (Bruker Micro-CT). The trabeculae were further analyzed by selecting a refined volume of 1 mm in height within the range of 0.5 mm distal to the growth plate of either femur or tibia. After applying a constant threshold of 80–255, BV/TV, Tb.N, Tb.Th, and Tb.Sp parameters were evaluated with CTAn program (Bruker Micro-CT).

### Resorption pit experiment.

The hydroxyapatite resorption experiment was conducted according to the previously described protocol (59). BMMs were seeded onto a 96-well hydroxyapatite-coated plate (3989, Corning Inc.) with 3 replicates. BMMs were treated with 50 ng/mL M-CSF and 25 ng/mL RANKL for a duration of 72 hours. Subsequently, each well was rinsed with a solution of 10% sodium hypochlorite to remove cells. The areas of the resorption pits were then captured using a microscope and quantified utilizing ImageJ software.

### Co-IP assay.

The HeLa cells were seeded onto 6 cm dishes and subjected to different treatments for 48 hours based on experimental assignments. Subsequently, the cells were immersed in lysis buffer supplemented with 1 mM PMSF, 1 mM dithiothreitol (DTT) (HY-15917, MCE), and a protease inhibitor cocktail. Immunoprecipitation was performed using Flag-tagged mouse monoclonal antibody (M1403-2, HUABIO; diluted at 1:100) and HA-tagged mouse monoclonal antibody (sc-7392, Santa Cruz Biotechnology Inc.; diluted at 1:100) overnight at 4°C. The resulting mixtures were then incubated with protein A/G beads for 3 hours at 4°C. Following 5 washes with PBS containing a protease inhibitor cocktail at 4°C, the beads were resolved using a 10% SDS buffer and subsequently analyzed by Western blotting.

### OVX-induced osteoporosis model.

The OVX-induced bone-loss model was established based on a previously described protocol (60). Briefly, 12-week-old female C57BL/6 *WT* or *NDR2-*cKO mice were anesthetized and subjected to bilateral ovariectomy or sham surgery. For ULK1-IN2 treatment, the WT OVX mice were randomly divided into 2 groups. The ULK1-IN2–treated group received i.p. injections of ULK1-IN2 at a dose of 0.5 mg/kg 3 times per week, while the control OVX group received PBS instead. After 8 weeks of model establishment, all mice were euthanized, and their femurs and tibias were collected and fixed in 4% paraformaldehyde for μCT scanning and bone histomorphometric analysis.

### Statistics.

At least 3 independent sets of experiments were conducted in triplicate, and the results were presented as mean ± SD. Statistical analyses were performed using Graph Pad Prism 9.0 for Windows (Graph Pad Software). In the statistical analysis, 1-way ANOVA and 2-way ANOVA were employed for multiple-comparison test. Unpaired 2-tailed Student’s *t* tests were utilized to compare means between 2 groups. Significance was determined at *P* < 0.05. *P < 0.05, **P < 0.01, ***P < 0.001, and ****P < 0.0001.

### Study approval.

All animal studies were approved by the Ethics Committee of Zhejiang University School of Medicine. All human studies were approved by the Ethical Review Board of Sir Run Run Shaw Hospital, Zhejiang University School of Medicine.

### Data availability.

Values for all data points in graphs are reported in the Supporting Data Values file.

## Author contributions

XK, ZS, YZ, ST, and Jingyun Chen performed the experiments, analyzed the data, and edited the manuscript. XK, ZJ, and JJ performed mice experiments and revised the manuscript. JL, WL, XJW, and JW conceived the project and revised the manuscript. FZ, BH, and Jian Chen conceived the project, designed the experiments, and edited the manuscript. All authors discussed the results and contributed to the preparation of the manuscript. Order of co–first authorship was based on the fact that XK conceptualized experiments and contributed validation, investigations, data visualization, and writing; ZS contributed investigations (data collection and experiment design) and editing; and YZ contributed literature search, methodology, and editing.

## Supplementary Material

Supplemental data

Unedited blot and gel images

Supporting data values

## Figures and Tables

**Table 2:**
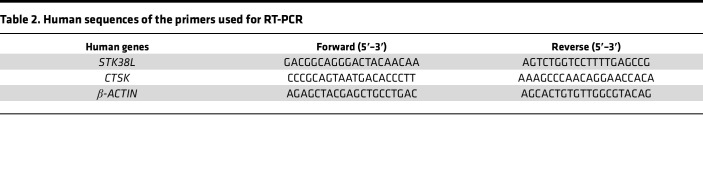
Human sequences of the primers used for RT-PCR

**Table 1:**
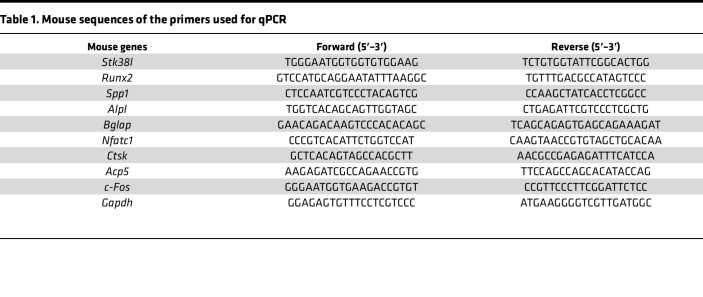
Mouse sequences of the primers used for qPCR

## Version 1 11/19/2024

In-Press Preview

## Version 2 01/09/2025

Electronic publication
