# Supplementary material for: NDR2 is critical for osteoclastogenesis by regulating ULK1-mediated mitophagy
Source: JCI Insight. 2024 Nov 19;10(1):e180409. doi: 10.1172/jci.insight.180409 (PMC11721311; doi:10.1172/jci.insight.180409)
Supplement: Supplemental data [file jciinsight-10-180409-s153.pdf]

NDR2 is critical for the osteoclastogenesis by regulating ULK1-mediated mitophagy

Xiangxi Kong[a,b#], Zhi Shan[a,b#], Yihao Zhao[a,b#], Siyue Tao[a,b], Jingyun Chen[c], Zhongyin Ji[a,b], Jiayan Jin[a,b], Junhui Liu[a,b], Wenlong Lin[d], Xiaojian Wang[d], Jian Wang[e], Fengdong Zhao[a,b,e*], Bao Huang[a,b*], and Jian Chen[a,b,e*]

[a]Department of Orthopaedic Surgery, Sir Run Run Shaw Hospital, Zhejiang University School of Medicine, No. 3, Qingchun Road East, Hangzhou, 310016, P.R. China.
[b]Key Laboratory of Musculoskeletal System Degeneration and Regeneration Translational Research of Zhejiang Province, No. 3, Qingchun Road East, Hangzhou, 310016, P.R. China.
[c]Department of General Surgery, Sir Run Run Shaw Hospital, Zhejiang University School of Medicine, No. 3, Qingchun Road East, Hangzhou, 310016, P.R. China.
[d]Institute of Immunology and Department of Orthopedic Surgery, The Second Affiliated Hospital, Zhejiang University School of Medicine, Hangzhou, 310016, P.R. China.
[e]Department of Wound Healing, The First Affiliated Hospital of Wenzhou Medical University, Wenzhou, 325000, P.R. China.

# Xiangxi Kong, Zhi Shan and Yihao Zhao contributed equally to this work and should be regarded as the co-first authors.
* Fengdong Zhao, Bao Huang, and Jian Chen are co-corresponding authors.

Key words: NDR2; STK38L; ULK1; osteoclast; osteoporosis; mitophagy

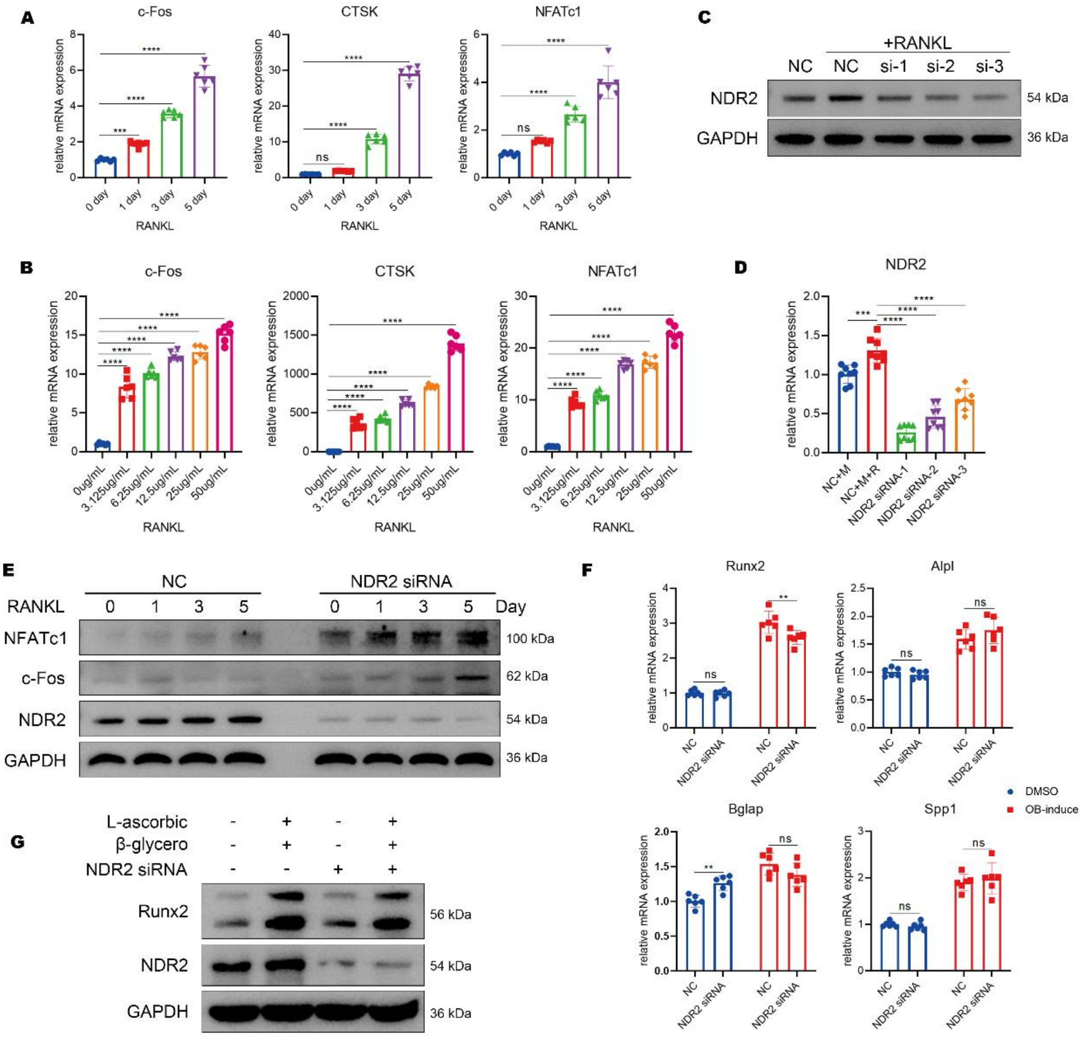

Figure.S1. NDR2 inhibited osteoclastogenesis. (**A**) The expression levels of osteoclast-related gene were assessed at various time points during osteoclast differentiation using RT-qPCR (n=6). (**B**) RT-qPCR was performed to assess the expression levels of the osteoclast-related gene after 48 hours of treatment with varying concentrations of RANKL (n=6). (**C**) Western blot and (**D**) RT-qPCR was utilized to assess the knockdown efficiency of NDR2 siRNA (n=6). (**E**) BMMs were transfected with NDR2 siRNA, and protein extraction was performed at different time points following RANKL induction. Western blot analysis was employed to determine the levels of osteoclast-related markers. (**F**) RT-qPCR was performed to assess the

expression levels of osteoblastogenesis-related genes (n=6). (**G**) Western blot was employed to determine the expression levels of osteoblast-related markers following NDR2 overexpression. The MC3T3-E1 cell line was transfected with NDR2 siRNA and subsequently induced for osteogenic differentiation using 5 μM ascorbic acid and 1 mM β-glycerophosphate. Statistical analyses were determined by one-way ANOVA (**A**, **B**, **D**) and two-way ANOVA (**F**). ns indicated no statistical difference, $**P<0.01$, $***P<0.001$, and $****P<0.0001$. Data were presented as mean ± SD.

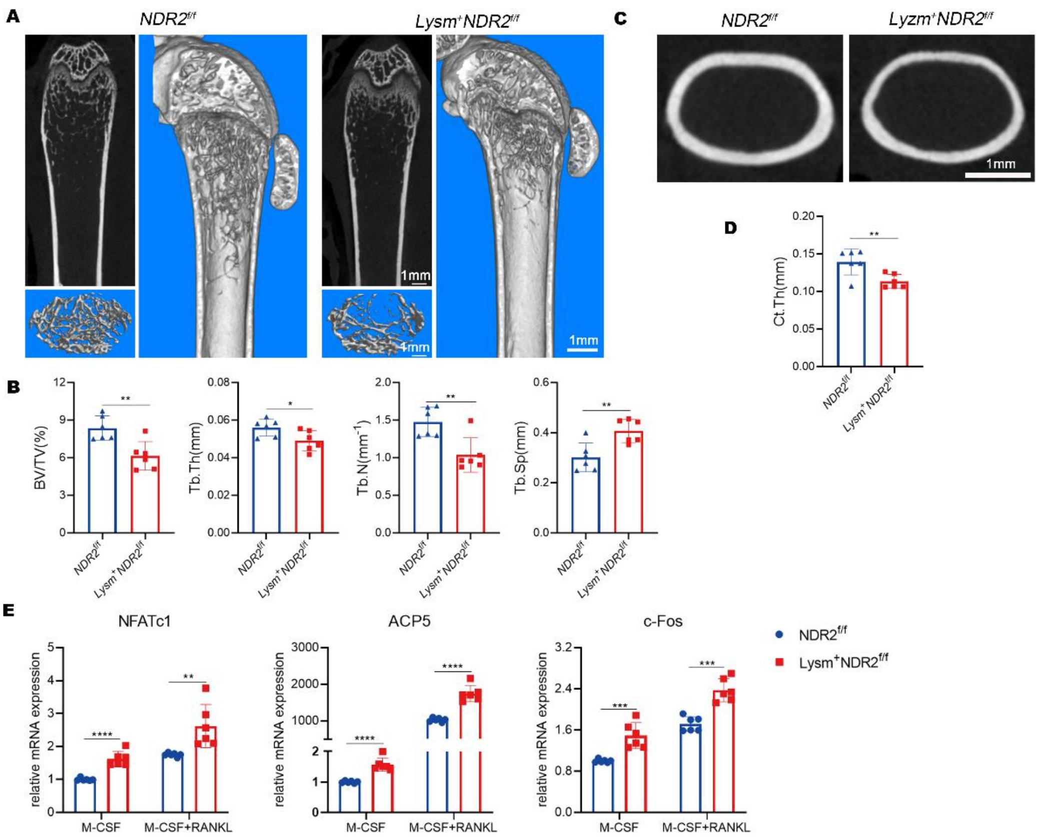

Figure.S2. *Lysm*$^{+}$*NDR2*$^{f/f}$ female mice exhibited decreased bone mass and attenuated osteogenesis. (**A**) Micro-CT images of femurs from 2-month-old female *Lysm*$^{-}$*NDR2*$^{f/f}$ and *Lysm*$^{+}$*NDR2*$^{f/f}$ littermates were obtained. (**B**) Trabecular bone parameters in the proximal femur (n=6) were assessed. (**C**) Represent two-dimensional images of cortical bone. (**D**) Statistical analysis was performed on Ct.Th (n=6). (**E**) Osteoclast-related gene levels were statistically analyzed in both *NDR2*$^{f/f}$ and *Lysm*$^{+}$*NDR2*$^{f/f}$ groups (n=6). Statistical analyses were determined by two-tailed Student's *t*-test (**B**, **D, E**). *P<0.05, **P<0.01, ***P<0.001, ****P<0.0001. Data were presented as mean ± SD.

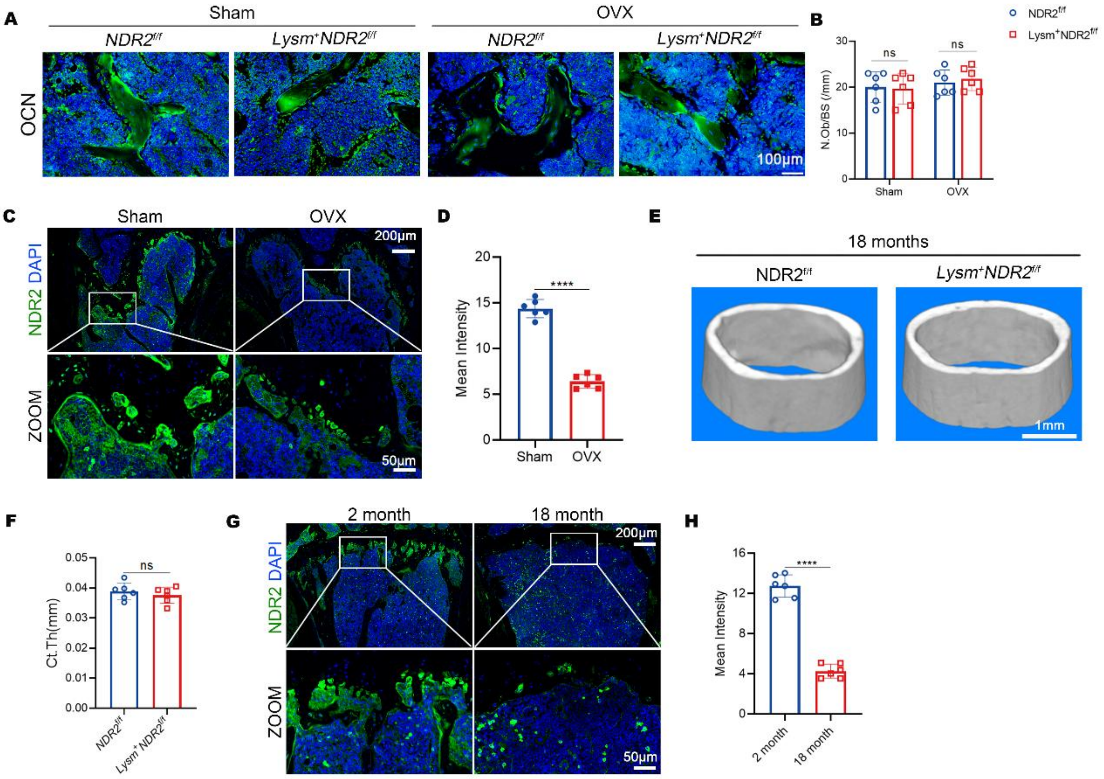

Figure.S3. ***Lysm^{+}NDR2$^{f/f}$*** **mice aggravated OVX-induced and aging-related bone loss.** (**A and B**) Representative images and quantification of OCN fluorescence staining of femoral sections. OCN (green), DAPI (blue). (**C** and **D**) Representative images and statistical analysis of NDR2 fluorescence staining of femoral slices from OVX and Sham groups (n=6). (**E**) Representative 3D images of cortical bone were captured. (**F**) Statistical analysis was performed on Ct.Th (n=6). (**G**and **H**) Representative images and statistical analysis of NDR2 fluorescent staining of 2-and 18-month-old slices (n=6). Statistical analyses were determined by two-tailed Student's *t*-test (**D**, **F**, **H**) and two-tailed Student's *t*-test (**B**). ns indicated no statistical difference and ****P<0.0001. Data were presented as mean ± SD.

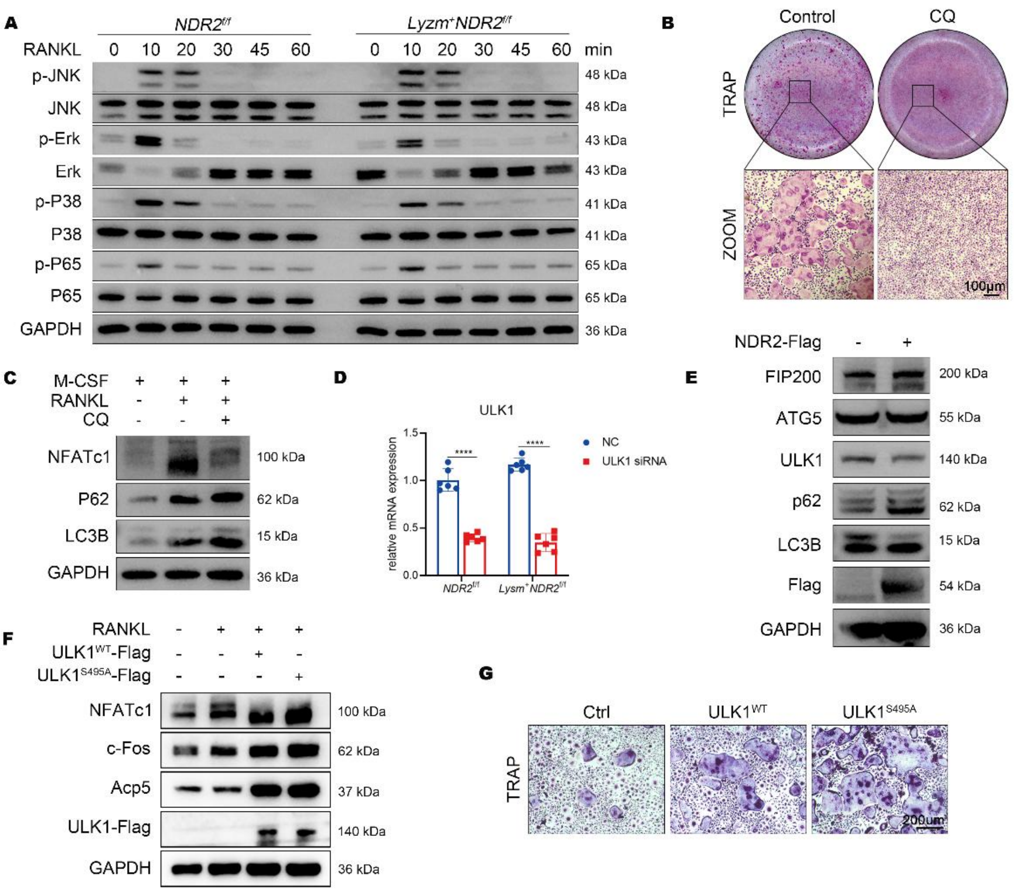

Figure.S4. NDR2 regulated osteoclastogenesis via ULK1. (**A**) Western blot of MAPK and NFκB signaling pathways. (**B**) TRAP staining of osteoclasts with or without CQ (20 μM). (**C**) Western blot of osteoclast-related indicators administered with or without CQ (20 μM). (**D**) RT-qPCR validation of ULK1 siRNA knockdown efficiency (n=6). (**E**) After BMMs were transfected with NDR2-Flag overexpression plasmid, the protein was extracted to detect the levels of autophagy-related proteins. (F) Western blotting was used to detect differences in expression levels of osteoclast-related proteins. (G) Representative images of TRAP staining. After infecting BMMs with ULK1^{WT} and ULK1^{S495A} lentiviruses for 24 hours, osteoclasts differentiation was subsequently induced with RANKL. Statistical analyses were determined by two-way ANOVA (**D**). ****P<0.0001. Data were presented as mean ±

SD.

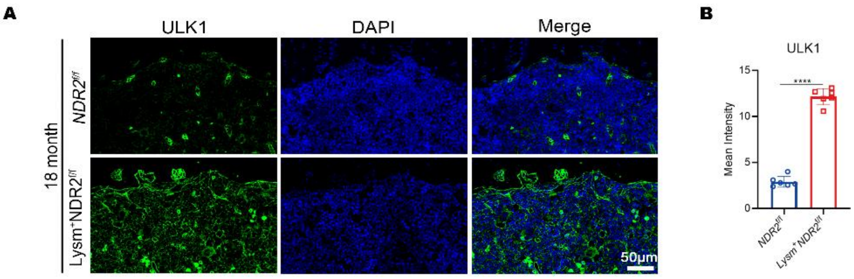

Figure.S5. NDR2 knockout enhanced ULK1 stabilization. Representative images (**A**) and statistical analysis (**B**) of ULK1 fluorescent staining of 18-month-old *NDR2$^{f/f}$* and *Lysm^{+}NDR2$^{f/f}$* groups slices (n=6). Statistical analyses were determined by two-tailed Student's *t*-test (**B**). ****P<0.0001. Data were presented as mean ± SD.

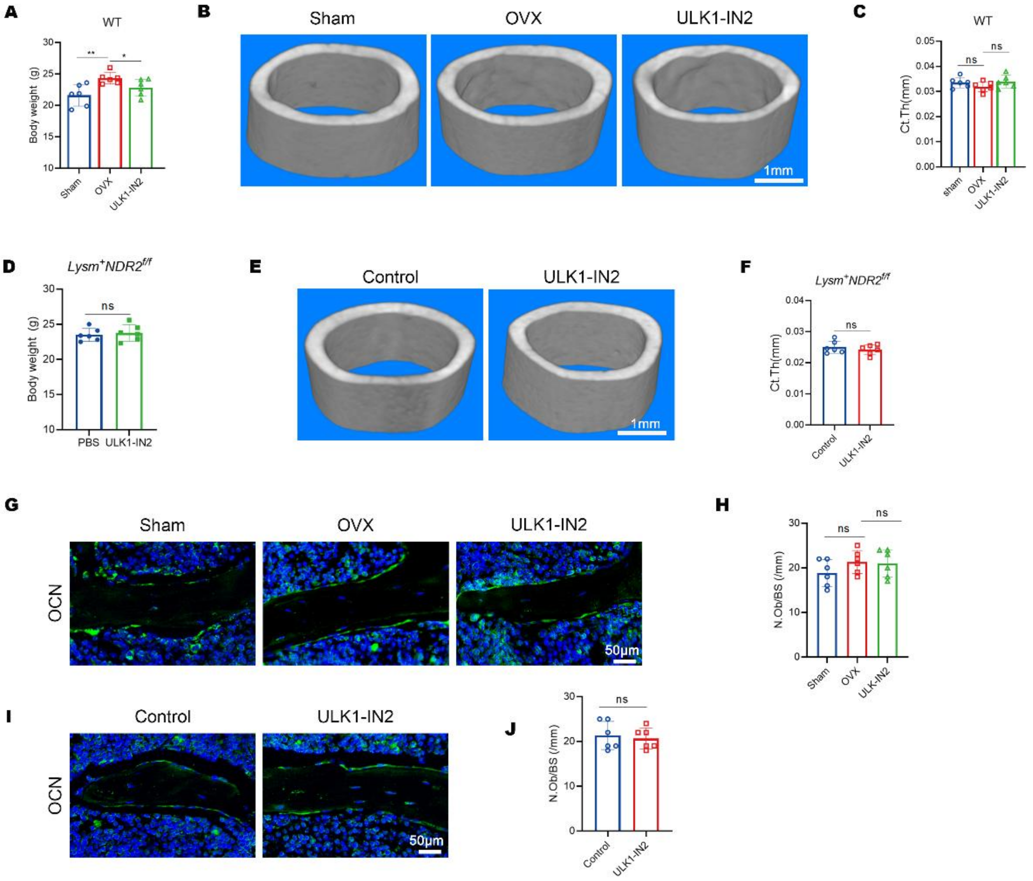

Figure.S6. ULK-IN2 rescued bone mass loss in both OVX model and *Lysm*$^{+}$*NDR2*$^{f/f}$ mice. (A) Statistical analysis of body weight of WT mice before sacrifice (n=6). (B) Representative 3D images of cortical bone were captured. (C) Statistical analysis was performed on Ct.Th (n=6). (D) Statistical analysis of body weight of *Lysm*$^{+}$*NDR2*$^{f/f}$ mice before sacrifice (n=6). (E) Representative 3D images of cortical bone were captured. (F) Statistical analysis was performed on Ct.Th (n=6). (G and I) Representative images of OCN fluorescence staining. (H and J) Quantification of osteoblast numbers based on OCN fluorescence staining. Statistical analyses were determined by two-tailed Student's *t*-test (D, F, J), one-way ANOVA (A, C, H). ns indicated no statistical difference, *P<0.05, **P<0.01. Data were presented as mean ± SD.

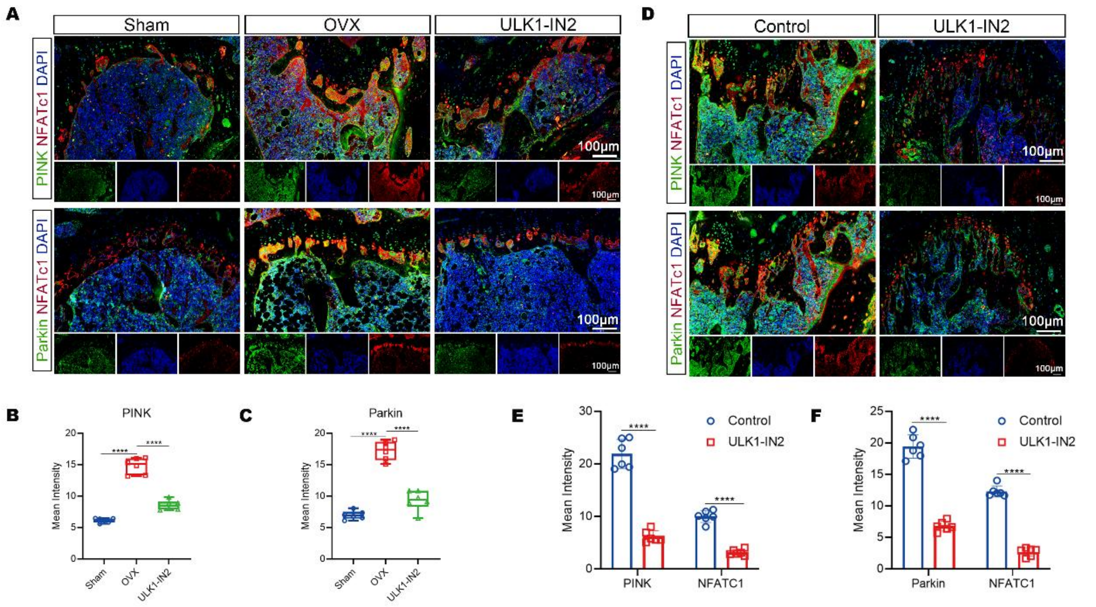

Figure.S7. Mitophagy was impaired by ULK-IN2 in both OVX model and *Lysm*$^{+}$*NDR2*$^{f/f}$ mice. (**A**) Immunofluorescence staining was performed on femur sections from OVX model with or without ULK1-IN2 administration to detect the expression of PINK and Parkin. The green fluorescence represents PINK/Parkin, while red fluorescence indicates NFATc1 (an osteoclast-related marker). Blue fluorescence marks DAPI. (**B** and **C**) Statistical analysis of fluorescence intensity (n=6). (**D**) Immunofluorescence staining was performed on femur sections from *Lysm*$^{+}$*NDR2*$^{f/f}$ mice with or without ULK1-IN2 administration to visualize the expression of PINK and Parkin. PINK/Parkin (Green), NFATc1 (red), and DAPI (blue). (**E** and **F**) Statistical analysis of the fluorescence intensity (n=6). Statistical analyses were determined by two-tailed Student's *t*-test (**E**, **F**), and one-way ANOVA (**B**, **C**). ns indicated no statistical difference, *P<0.05, **P<0.01, ***P<0.001, and ****P<0.0001. Data were presented as mean ± SD.
