# Supplementary material for: NDR2 is critical for osteoclastogenesis by regulating ULK1-mediated mitophagy
Source: JCI Insight. 2024 Nov 19;10(1):e180409. doi: 10.1172/jci.insight.180409 (PMC11721311; doi:10.1172/jci.insight.180409)

Full unedited gel for Figure 1A

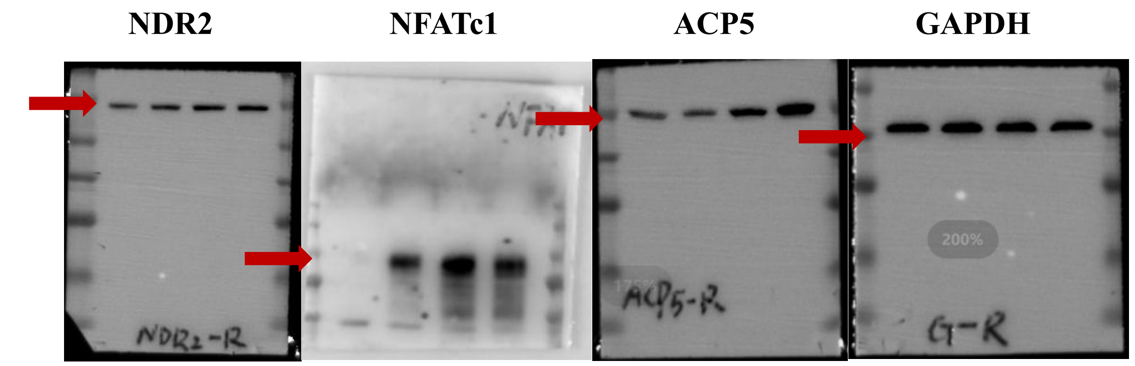

Full unedited gel for Figure 1C

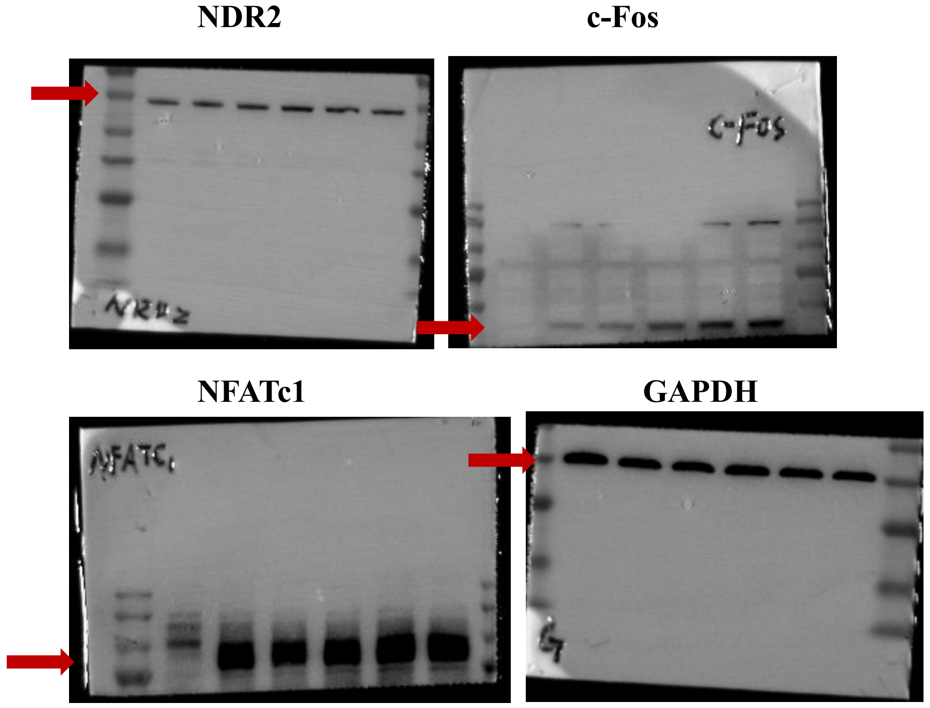

Full unedited gel for Figure 1J

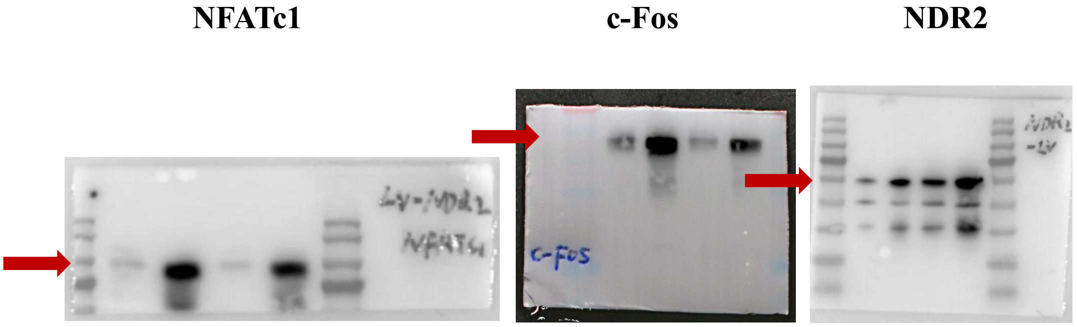

GAPDH

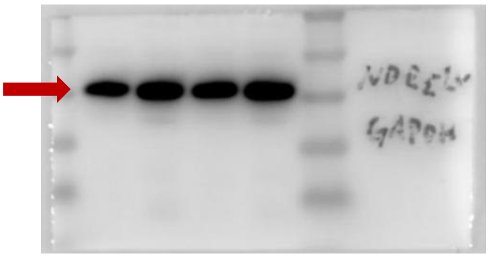

Full unedited gel for Figure 2I

NFATc1

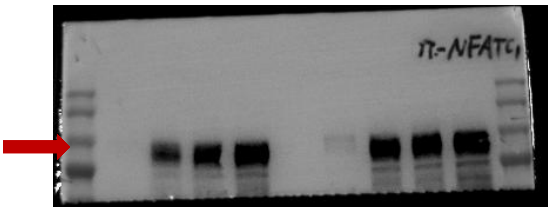

c-Fos

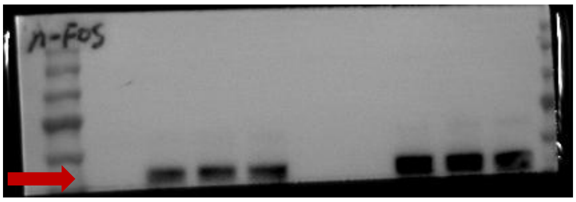

ACP5

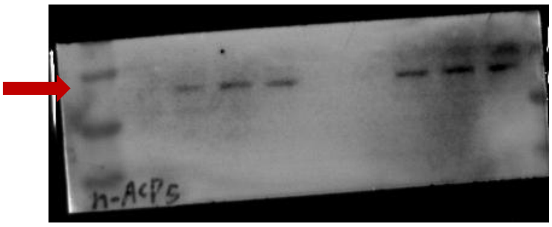

GAPDH

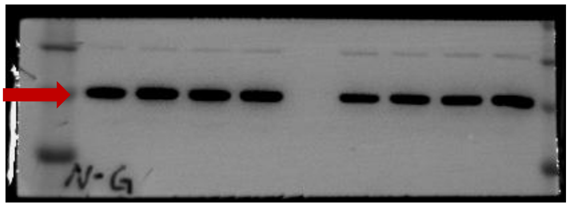

Full unedited gel for Figure 4B

ULK1

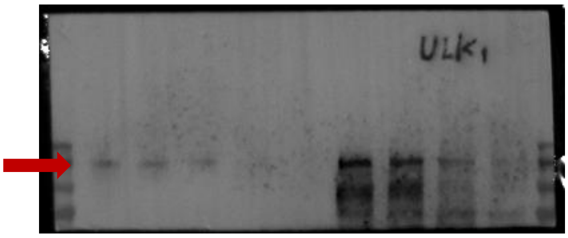

FIP200

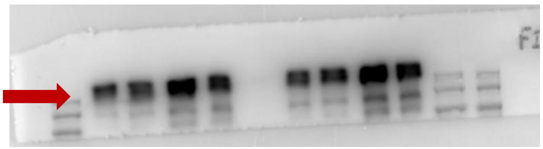

ATG13

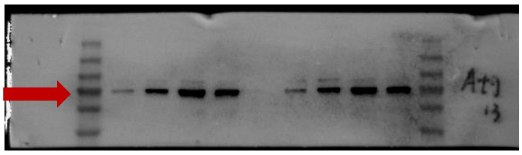

Beclin1

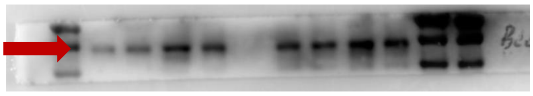

ATG12

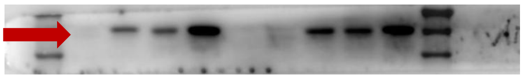

P62

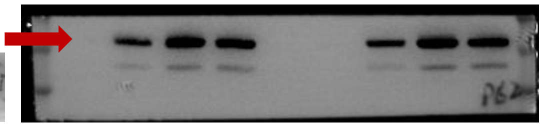

LC3B

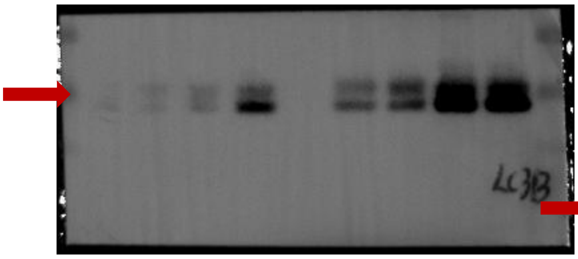

NDR2

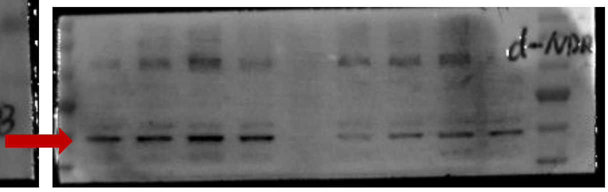

GAPDH

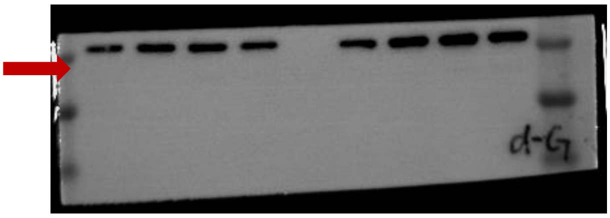

Full unedited gel for Figure 4C

NFATc1

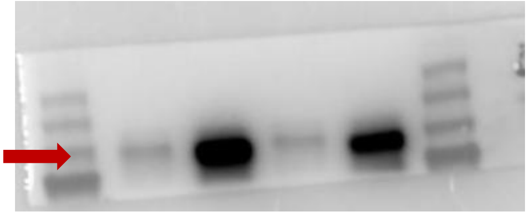

c-Fos

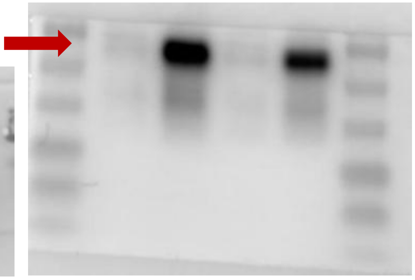

LC3B

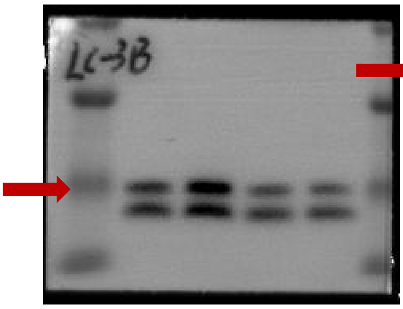

GAPDH

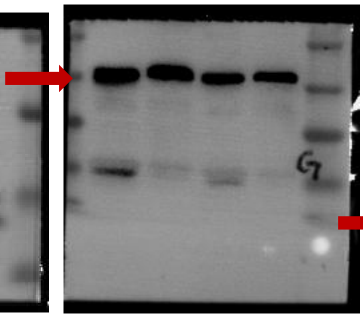

ULK1

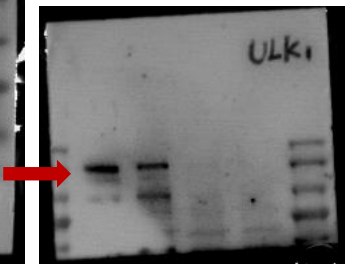

Full unedited gel for Figure 4F

NFATc1

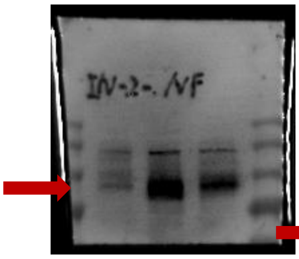

c-Fos

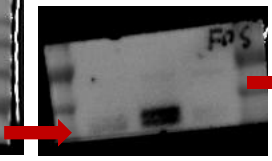

LC3B

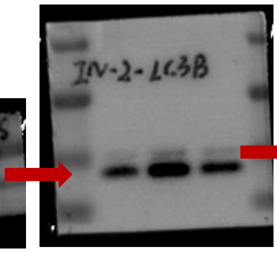

GAPDH

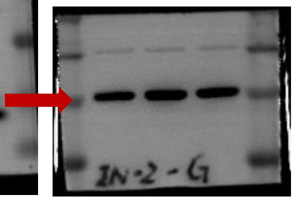

Full unedited gel for Figure 4G

NFATc1 c-Fos LC3B

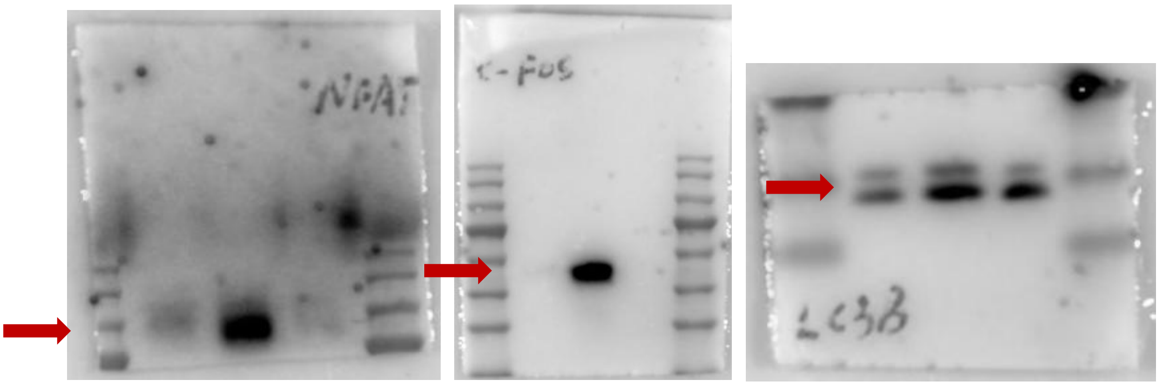

GAPDH

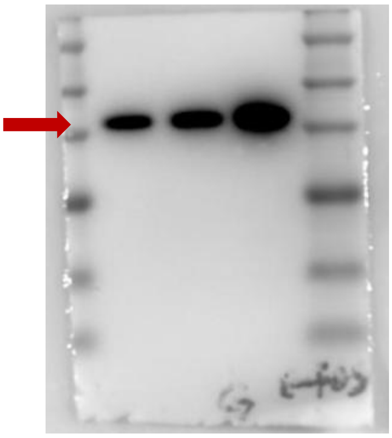

Full unedited gel for Figure 5E

ULK1 NDR2 GAPDH

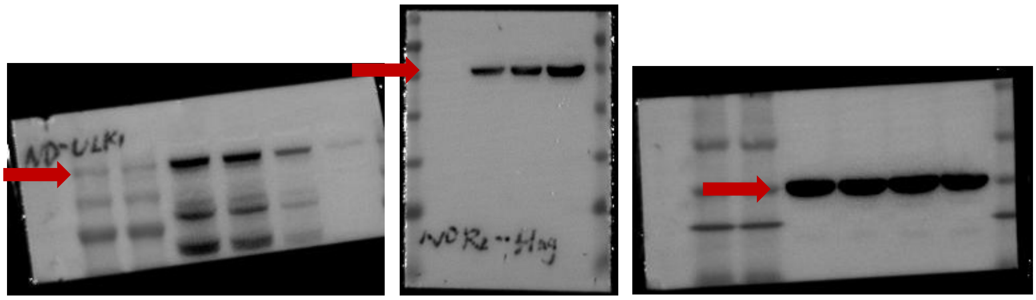

Full unedited gel for Figure 5F

IP:HA Flag IP:HA ULK1 IP:Flag ULK1 IP:Flag Flag

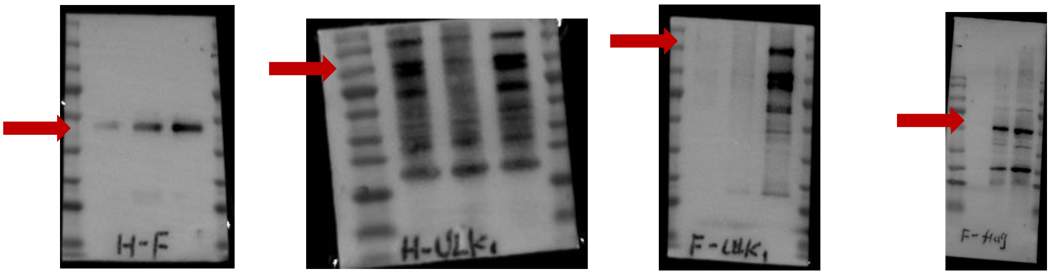

Input-Flag Input-ULK1 Input-GAPDH

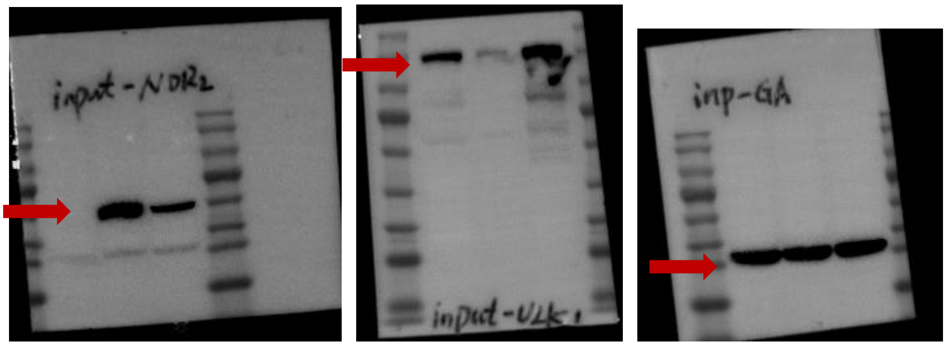

Full unedited gel for Figure 5G

ULK1 p-ULK1 NFATc1

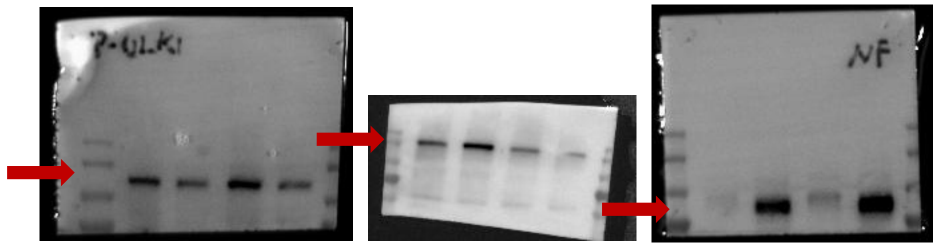

C-Fos NDR2 GAPDH

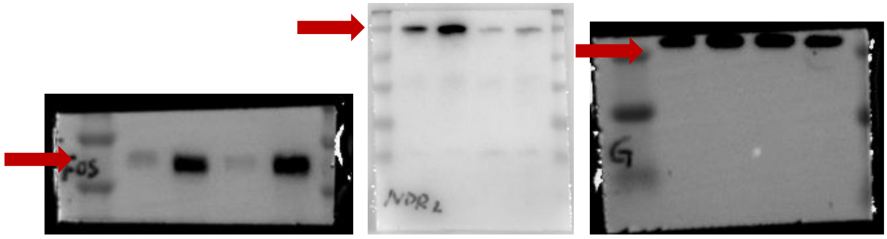

Full unedited gel for Figure 5H

IP-Ub IP-ULK1

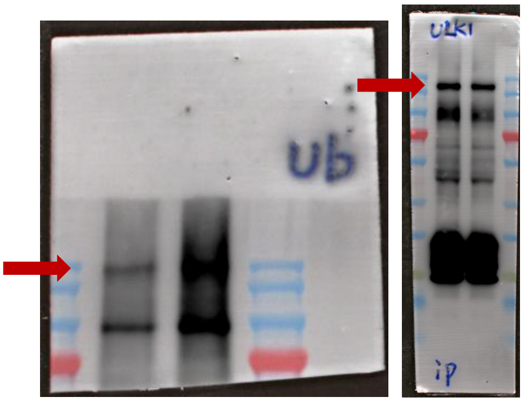

Input-ULK1 Input-NDR2-Flag GAPDH

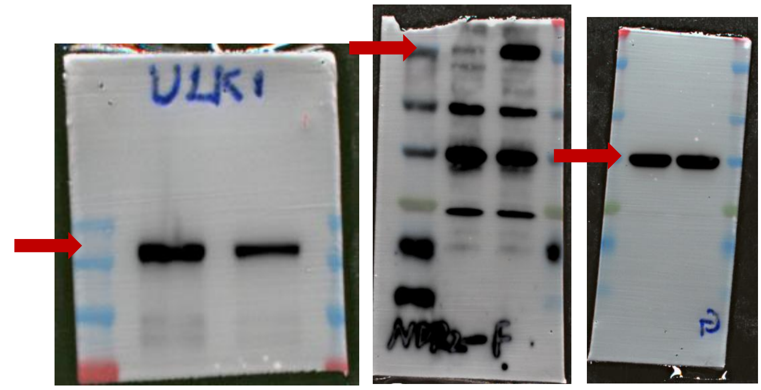

Full unedited gel for Figure 6B

Parkin PINK TOM20 c-Fos

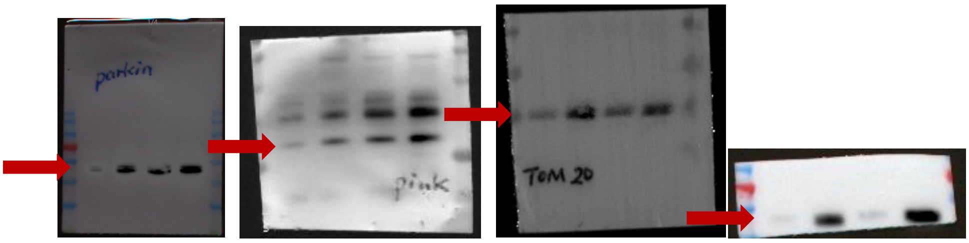

NDR2 GAPDH

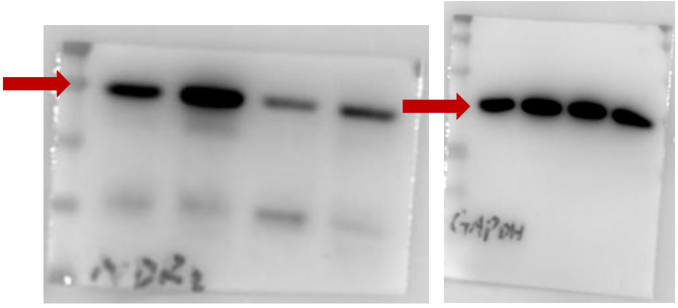

Full unedited gel for Figure 7F

PIINK Parkin TOM20 GAPDH

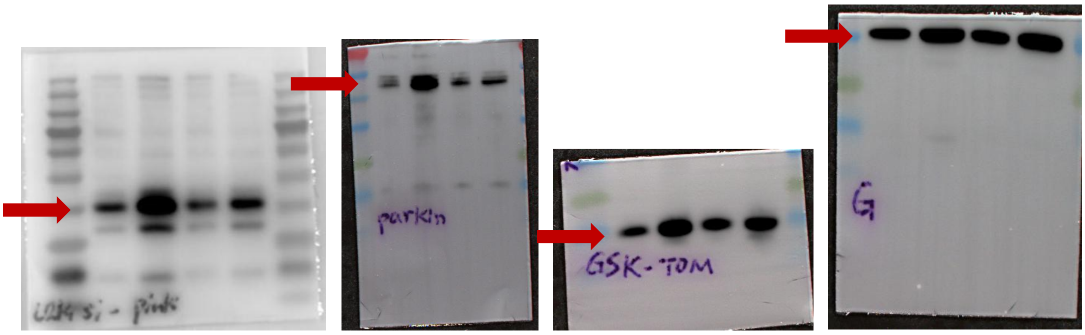

Full unedited gel for Figure 7G

Parkin PINK TOM20

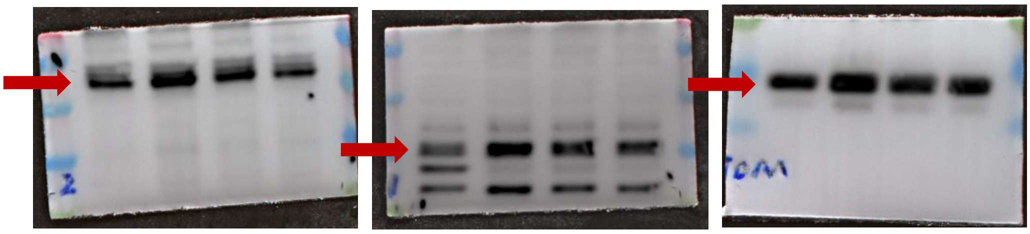

NFATc1 GAPDH

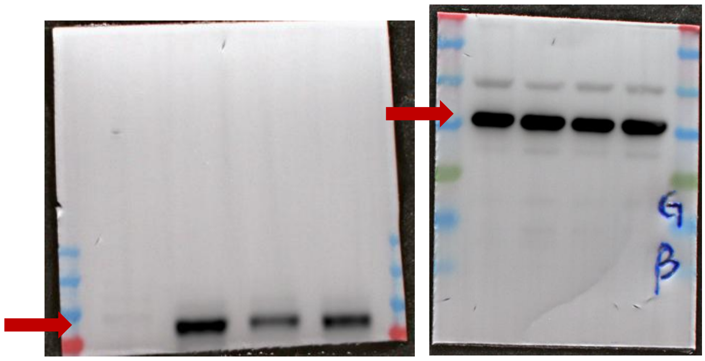

Full unedited gel for Figure 8D

ULK1 NDR2 GAPDH

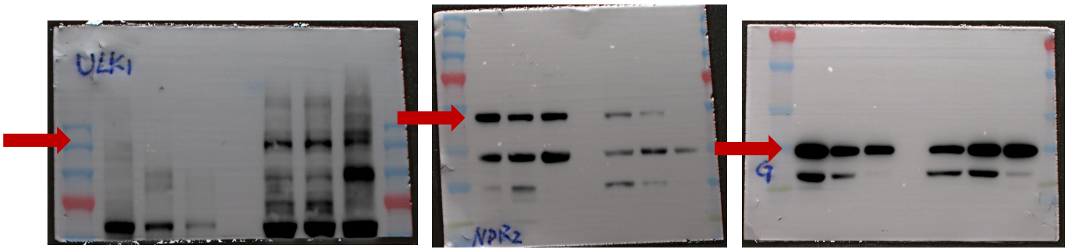

Full unedited gel for Figure S1C

NDR2

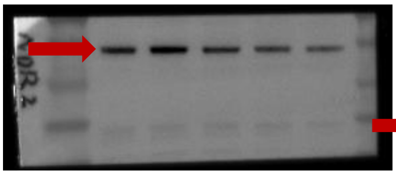

GAPDH

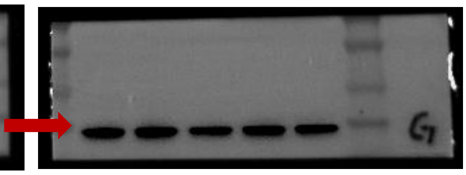

Full unedited gel for Figure S1E

NFATc1

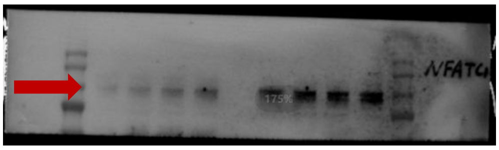

c-Fos

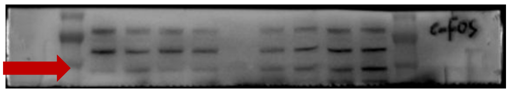

NDR2

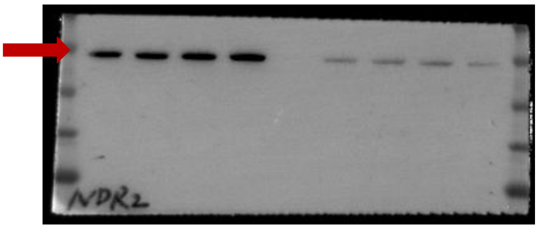

GAPDH

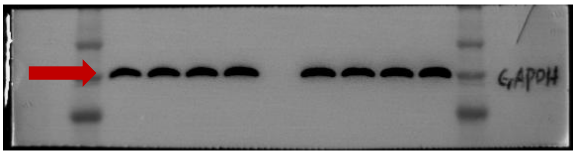

Full unedited gel for Figure S1G

GAPDH

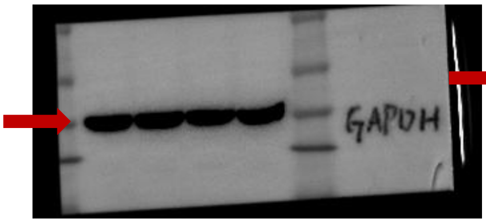

NDR2

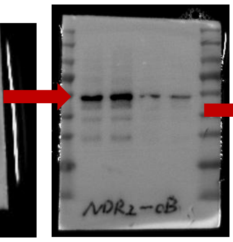

RUNX2

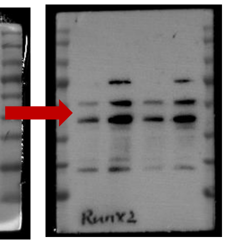

Full unedited gel for Figure S4A

p-JNK

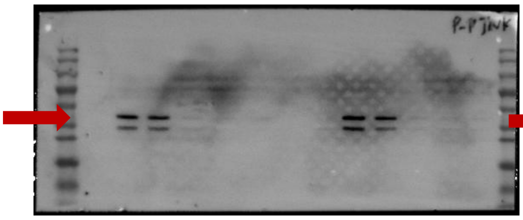

JNK

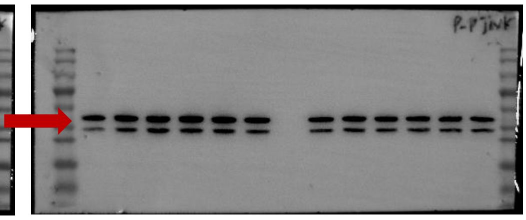

p-Erk

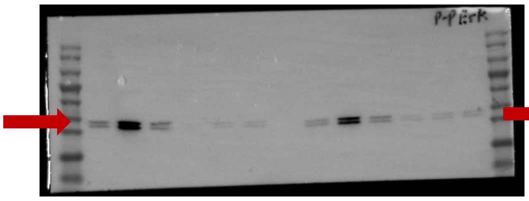

Erk

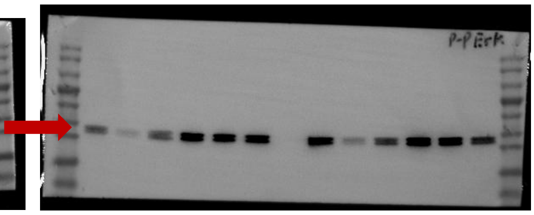

p-P38

P38

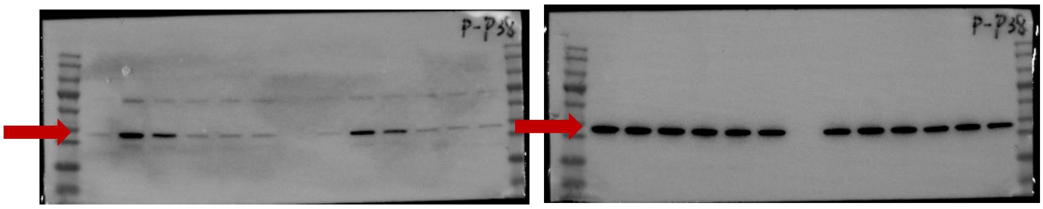

p-P65 P65

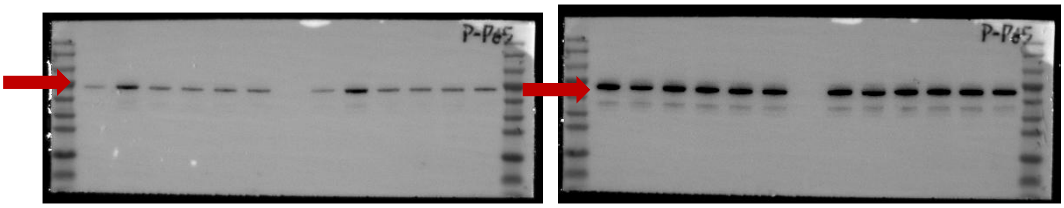

GAPDH

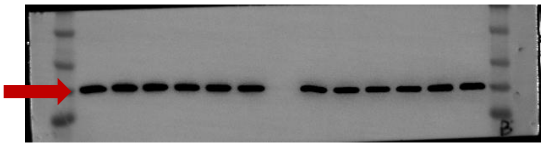

Full unedited gel for Figure S4C

NFATc1 p62 LC3B GAPDH

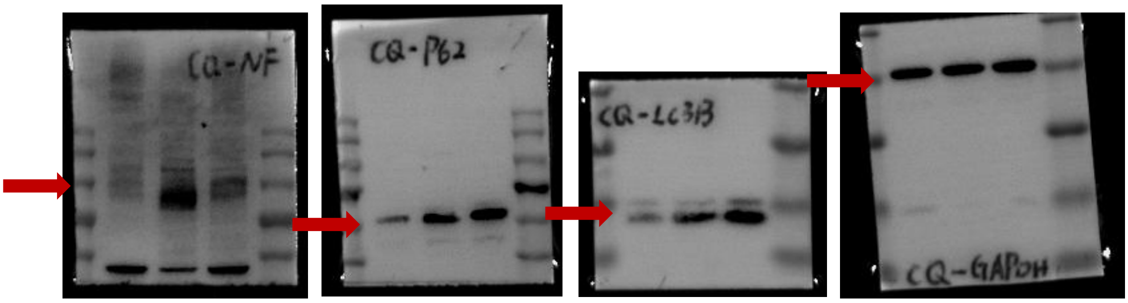

Full unedited gel for Figure S4E

FIP200 ATG5 ULK1 P62 LC3B Flag GAPDH

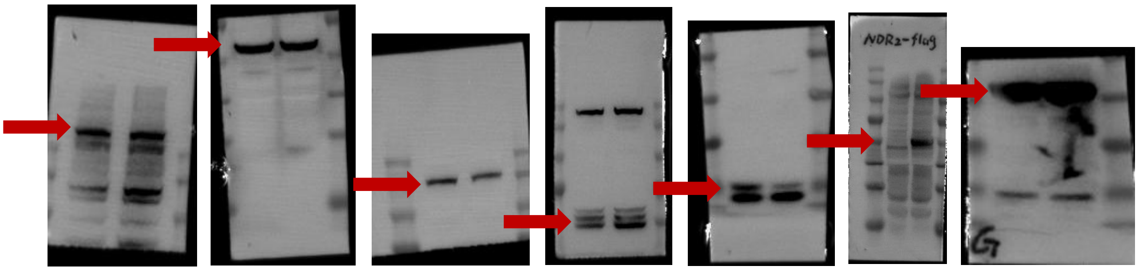

Full unedited gel for Figure S4F

NFATc1 c-Fos ACP5

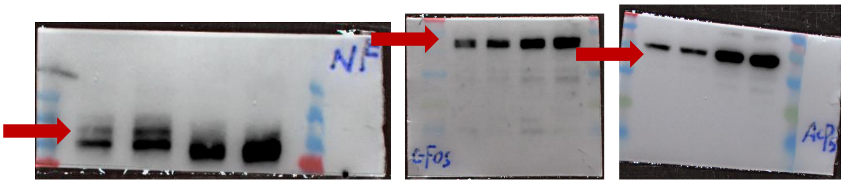

ULK1

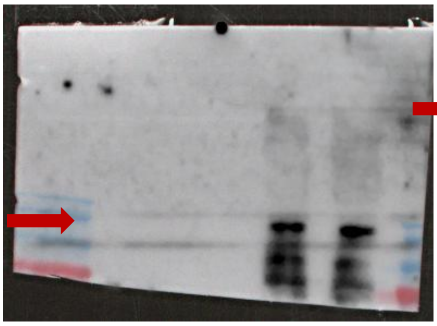

GAPDH

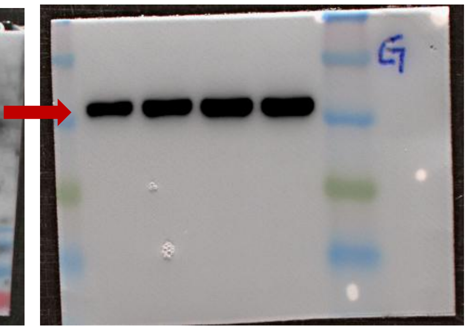

Supplement: Unedited blot and gel images [file jciinsight-10-180409-s154.pdf]
